# Integrating Artificial Intelligence into Circular Strategies for Plastic Recycling and Upcycling

**DOI:** 10.3390/polym18020306

**Published:** 2026-01-22

**Authors:** Allison Vianey Valle-Bravo, Carlos López González, Rosalía América González-Soto, Luz Arcelia García Serrano, Juan Antonio Carmona García, Emmanuel Flores-Huicochea

**Affiliations:** 1 CeProBi, Instituto Politécnico Nacional, Carr. Yautepec-Jojutla km. 6.5, Col. San Isidro, Yautepec 62739, Morelos, Mexico; avalleb2103@alumno.ipn.mx (A.V.V.-B.); clopezgo@ipn.mx (C.L.G.); rsoto@ipn.mx (R.A.G.-S.); 2 CIIEMAD, Instituto Politécnico Nacional, 30 de Junio de 1520 s/n, La Laguna Ticoman, Gustavo A. Madero, Ciudad de México 07340, Mexico; lugarcias@ipn.mx (L.A.G.S.); jcarmonag2400@alumno.ipn.mx (J.A.C.G.)

**Keywords:** circular plastic systems, artificial intelligence in recycling, chemical and biological depolymerization, upcycling of plastics, life cycle assessment, intelligent sorting technology

## Abstract

The increasing urgency to mitigate plastic pollution has accelerated the shift from linear manufacturing toward circular systems. This review synthesizes current advances in mechanical, chemical, biological, and upcycling pathways, emphasizing how artificial intelligence (AI) is reshaping decision-making, performance prediction, and system-level optimization. Intelligent sensing technologies—such as FTIR, Raman spectroscopy, hyperspectral imaging, and LIBS—combined with Machine Learning (ML) classifiers have improved material identification, reduced reject rates, and enhanced sorting precision. AI-assisted kinetic modeling, catalyst performance prediction, and enzyme design tools have improved process intensification for pyrolysis, solvolysis, depolymerization, and biocatalysis. Life Cycle Assessment (LCA)-integrated datasets reveal that environmental benefits depend strongly on functional-unit selection, energy decarbonization, and substitution factors rather than mass-based comparisons alone. Case studies across Europe, Latin America, and Asia show that digital traceability, Extended Producer Responsibility (EPR), and full-system costing are pivotal to robust circular outcomes. Upcycling strategies increasingly generate high-value materials and composites, supported by digital twins and surrogate models. Collectively, evidence indicates that AI moves from supportive instrumentation to a structural enabler of transparency, performance assurance, and predictive environmental planning. The convergence of AI-based design, standardized LCA frameworks, and inclusive governance emerges as a necessary foundation for scaling circular plastic systems sustainably.

## 1. Circular Strategies for Plastic: Concepts and Global Context

The circular economy (CE) represents a systemic shift from traditional linear production models toward regenerative cycles in which materials retain value for as long as possible. This transition relies on strategies such as reuse, repair, refurbishment, remanufacturing, and recycling, aiming to minimize waste generation and extend product lifetimes through multiple value loops [1]. In plastics, the relevance of circularity becomes particularly evident when considering the material’s history: early biobased materials (e.g., natural rubber and shellac) were progressively replaced by synthetic polymers that, during and after the Second World War, were engineered to be lightweight, durable, hygienic, chemically resistant, and easily manufacturable at scale [2]. These advantages accelerated global demand and positioned plastics as indispensable in modern life, while simultaneously entrenching highly linear patterns of production and disposal.

Since the 1950s, plastic production has increased more than 230-fold, reaching nearly 400 Mt in 2022, while recycling rates remain below 10% and landfilling persists as the dominant waste pathway in many regions [3]. While plastic pollution is often associated with post-consumer residues, a substantial share of plastic volume accumulates before products even reach use. Industrial surpluses, idle inventories, and stockpiled goods contribute to long-term material accumulation, creating a hidden reservoir of future waste that is rarely considered in conventional waste models. As shown by Geyer et al. [4] in 2015, 105 Mt of plastics entered global stock without fulfilling their intended functions, highlighting the need for early recovery strategies, improved resource stewardship, and stronger traceability across supply chains.

The scale of the challenge is amplified by the dominance of a limited set of polymers—PE, PP, PET, PVC, PS, HDPE, and LDPE—across packaging, construction, textiles, automotive, and electronics. Regulatory and societal drivers are increasingly shaping circular strategies for automotive plastics. Vehicles are long-lived, high-volume products whose growing polymer content aligns with public expectations for sustainable mobility, climate objectives, and resource-security concerns. Societal drivers are increasingly shaping circular strategies for automotive plastics.

These societal and policy pressures have increasingly translated into regulatory “market-pull” instruments that reshape circular priorities in downstream manufacturing sectors. In the European Union, the ongoing revision of end-of-life vehicle (ELV) rules has been framed not only as a waste-management measure but also as a circularity and traceability intervention responding to public expectations, climate commitments, and concerns about material security and value leakage. Within this context, policymakers have advanced mandatory minimum recycled plastic content targets for new vehicles (including a 25% target under discussion in the legislative process, with intermediate targets and provisions to promote closed-loop recycling), aiming to create stable demand for high-quality recyclates and incentivize investment in advanced sorting, decontamination, and quality-assurance infrastructures. This example illustrates that circular strategies are shaped by human lifestyles and consumption patterns—such as mobility demand and product longevity—alongside governance mechanisms that determine which circular pathways are feasible at scale. While automotive initiatives highlight the role of policy-driven demand for high-specification recyclates, the most significant mass flows and most immediate circularity bottlenecks remain concentrated in packaging streams, where material complexity and contamination frequently constrain mechanical recovery [5,6].

The packaging sector alone accounts for ~40% of annual production, but multilayer structures, pigments, additives, and volatile compounds significantly hinder recyclability and contaminate material streams, reducing the effectiveness and economics of mechanical recovery [3,7,8]. In parallel, environmental and health risks add another layer of complexity [9]. Plastic pollution affects terrestrial and aquatic environments, and microplastics and nanoplastics raise concerns due to their mobility, bioaccumulation potential, and capacity to transport harmful additives or absorbed toxins [9,10]. Degradation processes—including thermal oxidation, UV-induced fragmentation, and biological weathering—can also release greenhouse gases such as CO_2_, linking plastic mismanagement to climate change [8,11]. These considerations reinforce the need to expand the focus on circularity from end-of-life management to full-life-cycle optimization, encompassing product design, material selection, performance during use, and emissions control throughout the system [12].

Circularity in plastics is commonly conceptualized through circulation loops that define how value can be retained over multiple cycles. As outlined by Bucknall [3] and the Ellen MacArthur Foundation [13], these loops range from prevention through ecodesign and reuse, to mechanical recycling, upcycling, and chemical or biological depolymerization, with energy recovery as a last resort. Because each loop requires specific interventions, infrastructure, and quality thresholds, it is essential to understand their interactions and limitations before examining contemporary CE models or proposing new technological strategies.

From an operational standpoint, “plastic waste” is not a single feedstock but a spectrum of streams with distinct compositions, contamination levels, and degradation histories that directly constrain sorting performance, recycling yields, and product quality. Here, we propose a pragmatic classification by origin and complexity: (i) pre-consumer industrial scrap (narrow polymer distribution, low contamination) [14]; (ii) post-consumer packaging (high volume, high heterogeneity, frequent multilayers and additives) [15]; (iii) WEEE/ELV plastics (waste electrical and electronic equipment/end-of-life vehicles; engineering polymers, flame retardants, fillers, legacy additives) [16]; (iv) construction/demolition plastics (weathered, filled, mixed with inorganics) [17]. Across all classes, four feedstock descriptors should be reported where possible: polymer mix, format (rigid vs. film/flexible), contamination (food/soil/labels/moisture), and degradation/additive fingerprint (oxidation, pigments, fillers, stabilizers). These descriptors define the feasible circular pathway (mechanical, chemical, or biological) and the realistic substitution potential of recyclates. More broadly, the tendency toward quality loss across repeated cycles motivates tiered recycling strategies and the systematic use of LCA to evaluate trade-offs across pathways and contexts [18].

Within this evolving landscape, emerging digital technologies—particularly AI—are rapidly transforming circularity practices by enabling data-driven decisions across the plastic value chain. In this review, AI is considered in a functional sense as a set of methods (e.g., machine learning, computer vision, and optimization) that can improve identification and sorting, predict waste generation and material quality, optimize collection and logistics, and support scenario-based life-cycle modelling under uncertainty [19]. Recent work illustrates how coupling spectroscopy with ML can materially improve polymer identification under realistic, noisy conditions. For example, interpretable ML (dictionary learning) has been used to separate mixed FTIR signals and improve assignment robustness in microplastic analysis. Hyperspectral/portable NIR systems, supported by multivariate/ML models, enable near-real-time classification in field-relevant settings, although performance remains challenging for carbon-black plastics and thin transparent films. Complementary approaches such as Raman with AI-based preprocessing and LIBS with ML classifiers extend identification capability to heterogeneous and additive-rich streams, motivating the multi-sensor and sensor-fusion architectures reviewed in Section 2. Significantly, AI’s role is expanding from improving isolated tasks (e.g., classification or process control) toward supporting system-level decision frameworks that integrate material energy, regulatory, and economic constraints. Accordingly, rather than claiming that AI “determines” viability, we argue that AI can increasingly inform and optimize route selection and deployment conditions when coupled with robust sensing, reliable datasets, and transparent governance. This perspective frames the remainder of the review, which examines how AI-enabled processes and AI-enabled systems design can be evaluated and scaled under real-world constraints [19].

### Phase Evolution of AI in Circular Plastic System: From Instrument Support to System Enabler

In circular plastic systems, AI has evolved from improving isolated analytical steps to enabling higher-level system coordination. To clarify this progression, we propose a phased view of AI’s role, which also frames the structure of this review.

Phase I—Instrument support (task-level assistance): AI is primarily used to enhance measurement interpretation and classification in constrained settings (e.g., supervised models for FTIR/NIR/Raman spectra, baseline correction, denoising, peak attribution, and particle recognition). The value proposition is improved speed and accuracy for polymer identification and sorting decisions at the unit-operation level.

Phase II—Process optimization (line-level control): AI expands to optimize operational parameters and quality outcomes across a process line (e.g., multi-sensor decision rules, contamination detection, dynamic thresholding, predictive maintenance, and yield/quality optimization under variable feedstock). Here, AI links sensing to actuation, improving throughput and reducing mis-sorts, rejects, and energy penalties.

Phase III—System orchestration (network-level routing): AI supports routing and planning across collection, sorting, and recycling networks by integrating heterogeneous data streams (material composition, degradation/additive fingerprints, logistics constraints, market specifications, and environmental metrics). In this phase, AI functions as a decision-support layer for choosing among mechanical, chemical, biological, and energy-recovery pathways under uncertainty, often coupled with scenario-based LCA/TEA.

Phase IV—Structural enabler (design and governance integration): AI contributes to system design by enabling continuous updating of circular strategies through feedback loops, digital traceability, and governance-aligned optimization. Rather than optimizing a fixed process, AI helps define which routes are feasible, where they should be deployed, and under what compliance and monitoring requirements. This phase requires reliable datasets, transparency, and robust governance to avoid bias, ensure accountability, and maintain trust in automated decisions.

This phased lens connects the review’s technical sections (advanced sensing and AI-enabled sorting) with broader system-level considerations (routing, techno-economic and environmental trade-offs, and policy/traceability constraints), ensuring that AI’s role is discussed consistently from instruments to infrastructure-scale circularity.

## 2. Advanced Sensing and Intelligent Sorting of Plastics

### 2.1. The Strategic Role of Intelligent Sorting in Circular Plastic Systems

Plastic waste classification is a decisive stage in circular valorization pathways because it determines the quality of recovered materials and, ultimately, the feasibility of reintegration into mechanical, chemical, and upcycling routes. These routes differ markedly in process complexity, energy demand, emissions profiles, and tolerance to feedstock heterogeneity. As summarized by Naeim et al. [20], plastic can be managed through mechanical, chemical, thermal, physical, and biological processes, each with specific advantages and operational constraints closely linked to polymer purity, degradation state, and contamination levels.

In practice, mechanical recycling is widely adopted due to its relative simplicity and cost advantages, yet it is susceptible to mixed streams, contamination, and cumulative thermal-mechanical degradation. Chemical routes (e.g., pyrolysis, solvolysis, gasification) can tolerate more complex or degraded inputs and may regenerate higher-purity intermediates, but typically require higher energy inputs and stricter operating control. Thermal routes prioritize energy recovery for residues that cannot be materially recycled and therefore occupy a lower position in circular hierarchies due to emissions and by-product management requirements. Physical approaches (e.g., grinding, selective dissolution) preserve the polymer backbone but generally require more homogeneous input materials. Biological routes (microbial or enzymatic) are attractive from an environmental perspective; however, slow kinetics and intense sensitivity to contaminants still limit large-scale deployment. This diversity of options reinforces a central point: appropriate classification and sorting are prerequisites for routing each stream toward its most compatible pathway.

The stream origin is equally important for situating recycling strategies within life-cycle conditions. During conversation and manufacturing, virgin polymers generate post-industrial (PI) waste (sprues, trimmings, start-up residues) that typically remains clean, homogeneous, and compositionally known, enabling reintegration into higher-value loops. By contrast, end-of-life materials become post-consumer (PC) waste, which enters management systems as heterogeneous streams with uncertain composition and frequent contamination by organic residues and non-polymeric materials (e.g., paper, mineral particles) [21]. Accordingly, PI vs. PC origin directly conditions feasible recycling routes and expected quality outcomes.

Recycling pathways are also commonly described in terms of increasing processing intensity: primary recycling is generally limited to clean, well-characterized streams (often PI) that can be reprocessed with minimal processing loss; secondary recycling refers to mechanical treatment of PC waste and remains viable only while material quality is acceptable; tertiary recycling relies on chemical conversion when polymers are degraded or mixed; and quaternary recycling is reserved for functions that cannot be recovered materially, where valorization is limited to controlled energy recovery (e.g., co-incineration or partial oxidation such as gasification) [22].

System constraints frequently push waste downward in this hierarchy. In many developing contexts, a limited collection and separation infrastructure—combined with socio-economic and demographic factors—results in waste entering recovery systems in more degraded states [23]. This challenge is compounded by the increasing complexity of plastic products, including additives, blends, and hazardous components that hinder efficient separation [24,25,26]. Additives such as flame retardants, phthalates, bisphenol A, and heavy metals (e.g., cadmium and lead) can reduce recyclate stability and limit safe reuse [27]. Likewise, multilayer architectures (e.g., carton-based laminates containing paperboard, aluminum, and polyethylene) are challenging to disassemble and often require specialized processing or are routed to lower-value recovery options depending on local capabilities [28,29].

Consequently, PC waste frequently appears as highly heterogeneous mixtures with variable contamination, underscoring the need for robust sensing and AI-enabled sorting systems capable of assigning streams to the most compatible valorization pathways. This requirement motivates the advanced sensing and intelligent sorting strategies discussed in the following sections.

### 2.2. FTIR Spectroscopy and AI-Enhanced Signal Separation

Fourier-transform Infrared Spectroscopy (FTIR) is a core technique for polymer identification, owing to characteristic absorption bands in the mid-infrared region (4000–400 cm^−1^) [30]. However, environmental samples often contain sediments, fibers, and organic residues, which can generate overlapping spectra. When microplastic particles are filtered, the membrane itself introduces a confounding spectral signature, further increasing assignment uncertainty.

Beyond these physicochemical interferences, automated FTIR-based identification remains strongly dependent on the availability of curated, representative spectral reference databases. The limited coverage of real-world polymers, additives, and degradation states in existing libraries has been identified as a critical bottleneck for robust classification and large-scale automation [31].

To address this challenge, Buauk et al. [32] applied dictionary learning, an interpretable ML technique that decomposes mixed spectra into “spectral atoms” representing filter and polymer contributions. This digital separation reconstructs clean polymer signals even at low concentrations or under noisy conditions. Unlike deep neural networks, dictionary learning retains chemical interpretability and provides transparency into classification decisions—an advantage in regulatory or industrial contexts. These capabilities position FTIR combined with AI as a powerful tool for automated microplastic identification and high-precision sorting.

Despite its high spectral resolution, FTIR deployment beyond laboratory environments remains constrained by instrument cost, system footprint, and sample preparation requirements. In response, FTIR microscopy and imaging approaches have been developed to enable semi-automated analysis of entire filter areas without prior visual sorting. However, the performance of FTIR imaging is strongly dependent on the measurement mode and sample configuration. Transmission-mode FTIR is generally required for reliable detection, as specular reflection modes yield poor results due to weak infrared reflection from polymer surfaces and refraction artifacts caused by irregular particle geometries. At the same time, transmission imaging imposes strict constraints on particle thickness and filter substrates, which must be infrared-transparent and mechanically stable [33].

### 2.3. NIR, Minuaturization, and Machine Learning- and AI-Enhanced Signal Separation

Near-infrared spectroscopy (NIR) has become essential for real-time sorting to device miniaturization, enabling its use outside laboratory environments and into industrial and field settings. Although portable NIR units sacrifice spectral resolution compared with laboratory-grade systems, ML methods partially compensate for incomplete or noisy signals, enabling accurate classification [34,35,36]

Lubongo et al. [35] demonstrate that hyperspectral NIR combined with multivariate models effectively discriminates polymers such as PE, PP, PET, and PLA. However, classification performance declines markedly for dark-colored plastics and highly heterogeneous streams. Complementary evaluations by van Hoorn et al. [34] evaluated low-cost handheld devices (e.g., Plastic Scanner) revealed that hardware constraints—such as gaps between emission bands, low-power light-emitting diodes, and limited detector sensitivity—frequently lead to misclassification despite the use of advanced algorithms. Quantitatively, laboratory-grade NIR systems achieved accuracies of ~97% mid-range devices reached ~93%, whereas low-cost instruments showed substantially lower performance, ~70%.

These findings confirm that algorithmic sophistication cannot compensate for severe hardware limitations; ML can significantly enhance classification when spectral quality is moderate. Improvements in illumination bandwidth sources and improved detector sensitivity could therefore elevate portable NIR to industry-grade performance, making consolidating ML-enhanced NIR a cornerstone of accessible intelligent sorting systems in environments characterized by high material variability.

A critical intrinsic limitation of NIR-based sorting arises from the difficulty in distinguishing dark and black plastics that contain carbon black, one of the most widely used additives for imparting black or gray coloration. Carbon black exhibits strong absorption across the visible, ultraviolet, and near-infrared regions due to its extended conjugated graphitic structure, resulting in very low reflectance and feature-poor NIR spectra. As a consequence, black plastics are broadly recognized as particularly challenging—sometimes effectively unidentifiable—using conventional NIR approaches. While ML models applied to hyperspectral NIR datasets can extract subtle statistical patterns, classification performance for carbon black–containing plastics remains highly sensitive to data quality and experimental conditions, limiting robustness and generalizability in real-world waste streams [37].

Beyond color effects, NIR performance can also deteriorate in thin films and highly transparent plastics, where reduced absorption contrast and a short optical path length yield weak or indistinct spectral features. These constraints highlight that NIR-based identification is fundamentally bounded by the physics of light–matter interaction, rather than solely by algorithmic capability. In this context, NIR is most effective when applied to relatively homogeneous, non-black plastic streams, while more complex or additive-rich residues may require complementary sensing strategies [37,38]. Taken together, these limitations underscore the need to integrate NIR within multi-sensor sorting architectures rather than relying on it as a standalone solution.

### 2.4. Raman Spectroscopy Supported by Preprocessing and AI Algorithms

Raman spectroscopy provides chemically specific vibrational fingerprints and is less affected by moisture or matrix complexity than NIR, making it a valuable tool for the heterogeneous or degraded plastics [9]. Fang et al. [7] evaluated three ML models—nearest neighbors, random forest, and artificial neural networks—trained on Raman spectra of standardized plastics. In their study, due to baseline drift and noise, fluorescence identification emerged as the principal challenge, leading to baseline drift and spectral noise that hindered the identification of polymers such as ABS, PET, POM, and PVA. However, preprocessing (smoothing, baseline correction, normalization) dramatically improved accuracy.

Among the evaluated algorithms, the nearest neighbors model delivered the best balance of accuracy and computational speed, achieving 100% accuracy in controlled tests with processing times of ~4 ms. Combining spectral peak areas with statistical descriptors further improved robustness. Collectively, these results demonstrate that AI-enabled Raman systems can deliver rapid, high-precision identification, supporting the allocation of plastics across mechanical, chemical, and upcycling loops [7,9,39,40].

Nevertheless, methodological studies have shown that although Raman microspectroscopy can provide information on fillers and pigments that is not always accessible by FTIR, exclusive reliance on Raman analysis may lead to misidentification in real samples, particularly for coated or paint-derived particles. Accordingly, combined Raman–FTIR workflows have been repeatedly proposed as complementary strategies to improve particle-level characterization. Moreover, while Raman offers substantial potential for automated analysis of microplastics directly on filter substrates, performance is strongly influenced by surface-associated biological contamination: fluorescence induced by biofilms or other organic residues can dominate the Raman signal and prevent particle identification if appropriate sample preparation is not applied, and robust automation further requires systems capable of ensuring optimal focus on each candidate particle [37].

Raman measurements can be dominated by surface and near-surface layers in reflective configurations, potentially biasing identification toward coatings or the outer layer in multilayer laminates. Accordingly, confocal Raman depth profiling or cross-sectional mapping (when feasible), and/or complementary sensing (e.g., FTIR or elemental fingerprinting), are recommended for multilayer or coated plastics to reduce layer-driven misclassification.

### 2.5. LIBS: Elemental Fingerprinting and AI for Complex Waste Stream

Laser-induced breakdown spectroscopy (LIBS) has gained prominence for sorting complex or contaminated plastics, particularly those from electrical and electronic waste, where flame retardants or inorganic fillers must be detected. LIBS generates microplasmas that emit atomic signatures, enabling rapid identification.

Das et al. [24] analyzed 1800 LIBS spectra from six resin categories. They demonstrated that support vector machines and multilayer perceptron neural networks achieved 92–96% accuracy, reaching >98% under dynamic conditions simulating conveyor belt operation. LIBS combined with AI is especially effective where vibrational techniques fail due to overlapping molecular signatures or heavy additive content. Its robustness under variable color, texture, and composition makes it a strategic technology for the most complex sorting scenarios in circular systems. These advances remain strongly dependent on the availability of curated, representative spectral databases, which continue to constitute a critical bottleneck for large-scale deployment.

**Figure 1:**
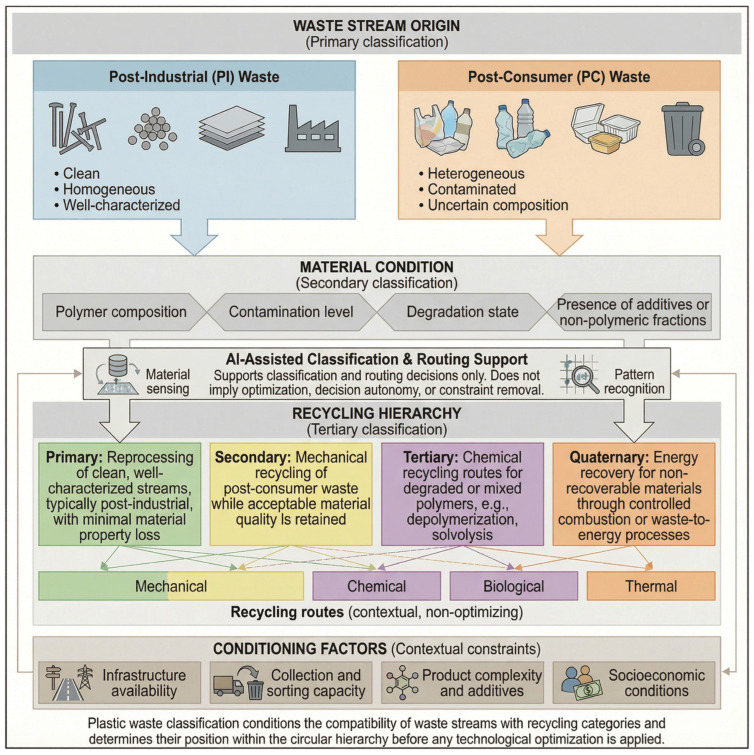
AI-enabled sensing and sorting framework for circular plastic systems. The diagram illustrates the integration of advanced sensing techniques (FTIR, NIR, Raman, LIBS, hyperspectral imaging) with ML algorithms for preprocessing, feature extraction, and classification. AI enhances identification accuracy, contamination detection, and routing decisions, enabling plastics to be directed toward the highest-value circular loops (mechanical recycling, chemical depolymerization, biological pathways, or upcycling). Arrows denote information/decision flow; lateral arrows indicate contextual inputs/constraints.

The key elements described across Section 2.1, Section 2.2, Section 2.3, Section 2.4 and Section 2.5 converge in a unified workflow, illustrated in Figure 1. This diagram illustrates how advanced sensing techniques combined with AI enhance accuracy, robustness, and efficiency in circular plastic sorting systems.

### 2.6. Emerging and Hybrid Modalities for Challenging Stream

Beyond conventional FTIR/NIR/Raman workflows, recently developed sensing approaches are increasingly being explored to address “hard cases” that routinely degrade sorting performance, including carbon-black plastics, highly transparent thin films, multilayer laminates, and streams with complex additive packages. In such cases, reliance on a single modality can be insufficient, as the limiting factor is often the underlying physics of signal generation (e.g., absorption and reflectance constraints) rather than algorithmic sophistication alone. Consequently, contemporary research and industrial pilots increasingly emphasize hybrid and multi-sensor architectures that combine complementary modalities and interpret them through AI-enabled sensor fusion [41,42].

A prominent direction is the use of mid-wave and long-wave infrared hyperspectral imaging (MWIR/LWIR-HSI) and other mid-infrared configurations, which can retain discriminative signatures for black plastics where VIS/NIR reflectance becomes feature-poor due to carbon black absorption. These approaches can be deployed as standalone classifiers or integrated with visible imaging, geometry cues, and probabilistic decision layers that quantify uncertainty and route ambiguous items toward secondary verification. In parallel, elemental and compositional fingerprinting (e.g., LIBS and related approaches) is increasingly viewed as essential for additive-rich streams (e.g., WEEE/ELV plastics, pigments, flame retardants), where polymer identification must be complemented with flags for halogens, fillers, and legacy additives to protect downstream processes and ensure compliance [43].

Another emerging direction is to reduce dependence on spectroscopic inference by embedding information carriers into products and packaging. Digital watermarking concepts and machine-readable identifiers can enable item-level identification when physical signatures are weak or confounded, providing a pathway to high-confidence routing for complex packaging formats. While such approaches require broad adoption and governance, they align with traceability-driven circularity strategies and can be coupled with automated sorting lines [44,45].

Finally, when biological or enzymatic routes are considered—particularly for streams originating from agricultural applications—feedstock qualification should include screening for pesticide/agrochemical residues and other inhibitory contaminants, as these can suppress biocatalytic performance and complicate downstream purification. Overall, these emerging modalities reinforce that advanced sorting should be treated as a multi-modal decision layer that integrates polymer identity, additive/degradation indicators, and uncertainty-aware routing to match each stream to the most compatible valorization pathway [46,47].

### 2.7. Data Availability, Database Development, and Governance Challenges for AI-Based Plastic Identification

The performance of AI-based systems for plastic identification is critically constrained by the availability, quality, structure, and governance of the spectroscopic data used for model training and validation. Although recent advances in ML and deep learning (DL) have demonstrated strong potential to automate polymer classification, the literature consistently shows that these models remain highly dependent on the data context in which they are developed and evaluated, limiting their generalization and transferability to real recycling scenarios.

Traditional chemometric and conventional ML approaches have demonstrated adequate performance under controlled laboratory conditions but are typically trained on relatively small, highly curated datasets. In this context, Tian et al. [48] demonstrated that classification performance is strongly influenced by dataset size, class imbalance, and spectral quality, showing that when datasets are small or imbalanced, even advanced models struggle to classify all polymer categories consistently. Importantly, these authors note that many published comparisons rely on datasets of fixed size, without systematically assessing how performance evolves as the number of samples changes—an important limitation given that real laboratory and industrial datasets are often constrained and heterogeneous.

From a complementary methodological perspective, Rosales-Martínez et al. [49] showed that, under controlled experimental conditions and with high-quality spectral data, the combination of appropriate spectral preprocessing—particularly derivative-based transformations—with ML models can yield extremely high classification accuracy for common polymers using FTIR. However, the authors explicitly caution that these results must be interpreted within their experimental context, as they are obtained from relatively pure and well-characterized samples. They further note that model generalization may be compromised by polymer mixtures, degradation, additives, contamination, or instrumental variability, which are characteristic of real PC plastic waste streams.

More recently, Singh et al. [50] demonstrated that advanced DL architectures, including Transformer-based models, can capture complex spectral patterns and outperform traditional chemometric and ML approaches across multiple spectroscopic datasets (FTIR, NIR, and hyperspectral NIR). Nevertheless, these authors emphasize that high performance is critically dependent on the availability of sufficiently large, diverse, and well-structured datasets, as well as on explicit control of data leakage and domain shift between experimental and industrial conditions. As a result, reported performances primarily reflect within-dataset generalization and do not guarantee direct transferability to unseen samples or operational sorting lines without additional adaptation strategies.

In addition to overall accuracy, AI-enabled sorting studies should report class-wise precision, recall, and F1-score (preferably macro-averaged), together with balanced accuracy and a confusion matrix, to avoid overstating performance in imbalanced datasets and to expose failure modes for “hard-case” streams (e.g., carbon-black plastics, thin transparent films, or additive-rich composites). Where probabilistic outputs are available, ROC–AUC/PR–AUC and calibration metrics can further support decision-threshold selection in safety- and compliance-relevant applications. Finally, reporting external validation (cross-site, cross-device, or temporally separated test sets) is recommended to quantify generalization under realistic distribution shifts and to ensure that gains observed in curated datasets translate into reliable performance in heterogeneous, real-world waste streams [15,51,52].

Taken together, these studies indicate that the primary bottleneck for the reliable implementation of AI-based plastic identification systems lies not in algorithm selection or model architecture, but in the development of robust, representative, and well-governed spectroscopic databases. In practice, most datasets reported in the literature originate from academic settings, are generated under controlled conditions, and lack the diversity required to reflect the intrinsic variability of post-consumer plastic waste, including different aging states, formulations, additives, contaminants, and acquisition conditions.

Beyond data availability, data governance emerges as a central challenge. The lack of standardization in spectral acquisition protocols, preprocessing pipelines, class labeling schemes, and dataset partitioning strategies limits cross-study comparability and model reproducibility. Both Singh et al. and Tian et al. [48,50] stress the need for more rigorous validation strategies, including grouped or site-aware data splits, formal statistical testing, and explicit evaluation of how dataset size and composition affect model performance.

Overall, current progress in building databases for AI-based plastic identification remains at an early stage. While public datasets and isolated expansion efforts exist, the reviewed literature consistently points to the need for larger, more diverse datasets, along with governance frameworks that ensure data traceability, quality control, and interoperability. These elements are essential to move beyond laboratory-scale proof-of-concept studies toward robust automated classification systems capable of operating reliably in real industrial and recycling environments.

### 2.8. Limitations and Future Challenges in AI Applications

Despite rapid advances, AI-enabled sensing and sorting in circular plastic systems faces several limitations that constrain real-world deployment and comparability across studies. Data limitations remain central: spectral and imaging datasets are often small, curated, and device-specific, with incomplete representation of real waste variability (additives, fillers, aging, contamination, multilayers). This leads to domain shift between laboratory conditions and operational environments, where changes in illumination, particle geometry, moisture, biofilms, and throughput can degrade performance. Class imbalance and ambiguous labels (e.g., blends, coated materials, carbon-black plastics, thin films) further complicate training and evaluation, underscoring the need for standardized reporting beyond accuracy (precision/recall/F1, confusion matrices, external validation) [41,42,43,48,49,50].

Operational constraints also limit adoption. Many sensing modalities require trade-offs among speed, footprint, sample preparation, and cost; moreover, mis-sorts may propagate downstream, affecting recyclate quality and safety. Robust deployment therefore requires closed-loop monitoring, routine performance verification, and integration with process logic (reject handling, re-routing, and quality gates). For high-stakes streams (e.g., WEEE/ELV fractions with legacy additives), AI outputs must be aligned with compliance needs and supported by confirmatory analytics and traceability [43,44,45].

Transparency and governance constitute additional challenges. Black-box models may be difficult to audit, and biased training data can systematically underperform on underrepresented waste fractions. To enable accountability, future work should prioritize interpretable models where feasible, clear confidence/decision thresholds tailored to application risk, and well-documented validation procedures. Shared benchmarks, open protocols, and harmonized metadata (polymer grade, additive/degradation fingerprints, device settings, environmental conditions) will be critical to improve reproducibility and transferability [48,50].

Looking forward, key opportunities include multi-sensor fusion (combining NIR/Raman/FTIR/LIBS with imaging) and traceability infrastructures (e.g., machine-readable identifiers/digital watermarking) that provide higher-quality upstream information. Finally, linking AI-enabled sorting with system-level decision support (LCA/TEA-informed routing) will be essential to ensure that performance gains translate into measurable circularity outcomes rather than local optimization [41,42,43,44,45].

## 3. Optimization of Mechanical, Chemical, and Biological Recycling Routes

Optimizing recycling routes within circular plastic systems requires addressing degradation pathways, improving material performance retention, and managing feedstock heterogeneity, as these conditions ultimately determine process viability, yield quality, and circular value recovery. Under CE principles, plastics are reincorporated into multiple-use cycles through mechanical, chemical, or biological routes, each of which requires tailored optimization strategies to increase recovery efficiency, reduce energy consumption, and sustain material performance across repeated loops. These recycling pathways present distinct operational boundaries driven by degradation history, contamination level, and polymer compatibility [29,53]. However, all are increasingly strengthened by emerging tools such as predictive modeling, catalyst-informed process design, and advanced bioengineering strategies that improve decision-making during sorting, processing, and final allocation.

### 3.1. Optimization of Mechanical Recycling

Mechanical recycling remains the most widely adopted and industrially implemented strategy due to its simplicity, low capital expenditure, and compatibility with existing infrastructure [3,54]. However, its performance strongly depends on the polymer history, contamination level, and the degree of thermo-oxidative degradation accumulated over the service life [21,22].

Recent advances demonstrate that process optimization begins at the extrusion stage. Edeleva et al. [53] report that control of residence time, screw configuration, melt rheology, and stabilizer dosing governs chain scission, crosslinking, and viscosity loss. When properly controlled, these variables improve the integrity of the recyclate and energy efficiency during extrusion.

Material compatibility is another key driver of optimization for mixed streams [55]. Hassanian-Moghaddam et al. [54] highlight that olefin-block compatibilizers, covalent-adaptable networks, and specific fillers expand the applicability of recycled polyolefins by improving interfacial adhesion. These stabilization strategies are especially relevant when transitioning from PI to heterogeneous PC flows. These approaches expand circularity thresholds for mixed polyolefin streams, often associated with packaging, automotive, and consumer goods waste [3,8].

Economically, mechanical recycling remains preferable when contamination is low and when feedstock quality supports high-value direct reuse. Uekert et al. [56] report that mechanical recycling outperforms other closed-loop options when sorting quality is adequate, and degradation remains below mechanical-property critical limits. Their analysis further indicates that the transition to chemical pathways occurs when the recyclate quality falls below the reprocessing grade specifications.

From a circular-strategy standpoint, low-input-energy material recycling (i.e., mechanical reprocessing into regranulates) should preferentially target products with high substitution potential and modest performance sensitivity, such as rigid packaging and non-food-contact consumer goods, as well as durable items (e.g., crates/pallets and construction profiles/pipes) where minor property drift can be accommodated. Conversely, high-specification applications (e.g., strict food-contact uses) generally require more stringent decontamination, traceability, and property assurance; when these conditions are not met, chemical or biological routes may become more appropriate to restore functionality at the monomer/intermediate level.

Moreover, integrating real-time monitoring with predictive algorithms enables decision-making before irreversible degradation occurs. Inline FTIR/NIR analysis, melt-flow index tracking, and residency-time prediction models allow assigning materials to appropriate loops before quality losses accumulate—improving yield retention and energy efficiency. Overall, mechanical optimization converges on:

- Minimizing thermo-mechanical degradation [8];
- Enhancing inter-polymer phase compatibility;
- Minimization of mass losses along washing, melting, and filtration steps;
- Integrating predictive extrusion-quality models.

### 3.2. Optimization of Chemical Recycling

Chemical routes—particularly pyrolysis, solvolysis, hydrogenolysis, and depolymerization—support recovery when waste is highly heterogeneous or degraded, making mechanical pathways unsuitable [20]. Catalyst engineering and the integration of kinetic models are central to improving purity-product curves.

Huang et al. [55] emphasize that process intensification through catalyst selection governs selectivity, hydrocarbon distribution, and coke formation. Catalyst–product relationships show that zeolites enhance aromatics, silica–alumina systems improve cracking balance, and fluid-catalytic designs accelerate conversion while reducing tar formation.

Life-cycle trade-offs relative to mechanical approaches have been quantified by Jeswani et al. [57], who conclude that environmental competitiveness improves only when thermal integration and selective upgrading steps are incorporated.

From an industrial perspective, Kumagai et al. [58] highlight three optimization priorities:

- Catalyst robustness across mixed streams;
- Reduction in energy intensity per ton converted;
- Validation of lab kinetic performance at pilot-plant scales.

Data-guided pyrolysis optimization is emerging as the most transformative area. Paavani et al. [59] and Tomme et al. [60] show that ML frameworks accurately predict wax, gas, aromatics, and fuel fractions based on input composition. These datasets establish predictive routes to maximize monomer-grade output while minimizing operating conditions via automated search spaces. A significant advance in the optimization of AI-enable thermochemical pathways is the study by Cheng et al. [61], which developed ML models to predict products from continuous non-catalytic pyrolysis of plastic waste accurately. To do this, they compiled a database derived from 93 experimental studies and evaluated four supervised algorithms: decision trees, artificial neural networks, support vector machines, and Gaussian processes. Its objective was to identify which variables allow the performance of solids, liquids, and gases to be predicted more accurately, as well as the specific compositions within each fraction. The results show that decision tree-based models far outperform the other approaches, achieving coefficients of determination greater than 0.99 for predicting waxes, aromatics, gasoline, light gases, and condensable fractions.

Collectively, optimization of chemical routes depends on:

- Kinetic-parameter predictive models;
- Catalyst recombination studies;
- Integrated heat-exchange schemes;
- Selective downstream purification.

### 3.3. Optimization of Biological Routes

Bacteria and fungi can fragment polymers under aerobic or anaerobic conditions, generating low-molecular-weight compounds that can be assimilated, integrated into native metabolic pathways, or further transformed into valuable products through metabolic engineering. These processes typically operate under mild conditions—near-ambient temperatures and neutral to moderately buffered pH—thereby reducing the energy requirements relative to thermochemical conversion routes [62,63]. Despite these advantages, the practical implementation of biological recycling remains limited mainly to polyester-based waste streams such as PET, due to inherent limitations in polymer accessibility and enzymatic specificity.

Enzymatic hydrolysis studies have demonstrated that PETases and MHETases can depolymerize PET into high-purity monomers, including terephthalic acid and ethylene glycol. However, high crystallinity and limited surface accessibility significantly constrain reaction kinetics, rendering depolymerization the rate-limiting step [10]. Consequently, preprocessing steps—e.g., amorphization, mechanical grinding, surface oxidation, or solvent-assisted swelling—are often required to enhance enzyme–substrate interactions and improve overall conversion efficiency.

Chen et al. [64] indicate that biologically driven valorization is most effective under hybrid schemes combining controlled oxidation followed by microbial conversion. An example is the study by Zhan et al. [65], who demonstrated that polyethylene, one of the most recalcitrant polymers due to the stability of its C–C bonds, can be chemically oxidized to generate C4–C6 dicarboxylic acids. A metabolically redesigned Corynebacterium glutamicum subsequently assimilated these intermediates. Similarly, Uekert et al. [56] further indicate that enzymatic conversion routes can become competitive when solvent volumes, wash-water demand, and enzyme loading are minimized through process integration and optimization.

Beyond polymer structure and pretreatment requirements, the feasibility of biological recycling routes is also conditioned by the chemical history of the waste stream. Recent studies have shown that polyethylene and polypropylene microplastics can adsorb a wide range of organic contaminants, including pesticides and pharmaceuticals, primarily through physical interactions such as van der Waals and electrostatic forces [66]. Field-based evidence further indicates that aged plastic fragments collected from agricultural environments retain measurable pesticide residues after real-use exposure, with concentrations in the polymer matrix often exceeding those detected in surrounding soils [67]. Taken together, these findings suggest that plastic materials used in agrochemical contexts may carry residual organic compounds beyond their service life. Such contamination not only constrains high-value upcycling applications but may also interfere with biological recycling routes, as residual agrochemicals can inhibit microbial metabolism or compromise enzymatic stability under the mild operating conditions required for biocatalytic conversion. Accordingly, biological valorization strategies require upstream screening and, where necessary, targeted decontamination steps to ensure compatibility between the waste stream and microbial or enzymatic systems.

A structural limitation nonetheless persists. In contrast to mechanical and chemical recycling, the optimization of biological routes is constrained by the limited availability of a curated enzymatic dataset, including:

- Comprehensive PETase and esterase mutational libraries,
- Kinetic profiles spanning different crystallinity grades and polymer morphologies;
- Systematic validation of ML-guided enzyme design workflows under industrially relevant conditions.

This data scarcity has historically slowed the translation of promising biocatalysts from laboratory discovery to scalable processes. Addressing this limitation, Jiang et al. [68] developed PEZy-Miner, a ML computational framework designed to identify enzymes with plastic-degrading potential from large sets of uncharacterized protein sequences. The approach combines protein language models that encode implicit structural and functional features with supervised classifiers that predict degradative activity across 11 polymer types. By prioritizing high-confidence candidates and quantifying prediction uncertainty, PEZy-Miner enables more efficient experimental screening and accelerates the discovery of novel biocatalysts, thereby supporting the development of modular and scalable biological recycling platforms.

### 3.4. Cross-Route Optimization Perspective

Across loops, optimization is influenced by feedstock state, compatibility requirements, and targeted product quality thresholds:

- Mechanical loops maximize value when polymer memory is known, and degradation is minimal.
- Chemical loops maximize value when the waste stream is heterogeneous, multilayered, or contaminated.
- Biological loops uniquely deliver monomer-grade purity but require substrate accessibility and bio-catalyst engineering.

At present, convergent industry trends emphasize integrating:

- ML-assisted condition prediction;
- TEA-LCA indicators;
- Quality-based routing logic.

This provides decision frameworks where each stream feeds into the most favorable loop based on circular-value recovery rather than cost alone.

### 3.5. Integrative Resources: Comparative Table and Conceptual Diagram

**Figure 2:**
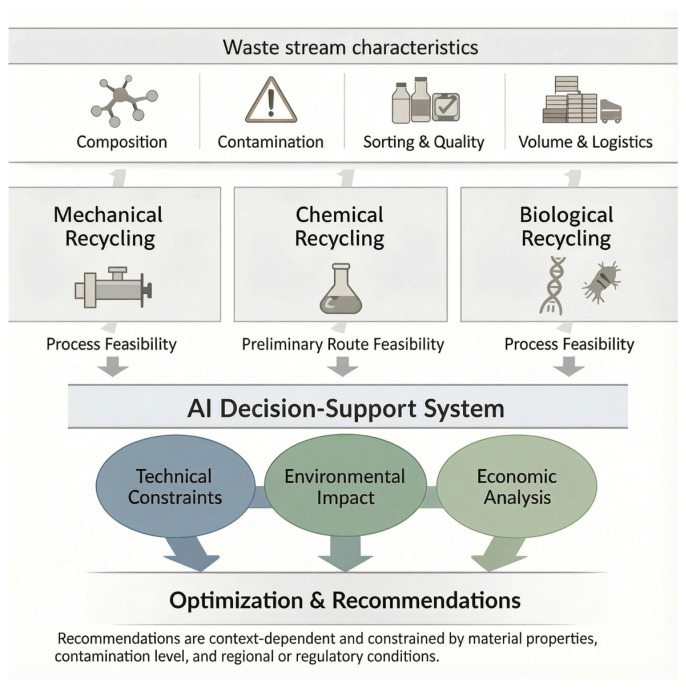
Comparative optimization routes in circular plastic processing.

To elucidate the interactions among optimization pathways within circular systems, Figure 2 presents a conceptual decision framework for allocating post-consumer and post-industrial plastics to mechanical, chemical, or biological recycling routes. Allocation criteria include material condition, processing constraints, and anticipated value retention. Complementarily, Table 1 provides a structured comparative summary of technological levers, quantitative descriptors, and performance indicators documented in the recent literature, enabling systematic evaluation of optimization priorities.

Importantly, route selection is not predetermined; it is informed by predictive decision-support models that integrate material quality, environmental impacts, techno-economic assessments, and digitally enabled diagnostics. Mechanical recycling performance is primarily governed by melt stability and compatibilization strategies, whereas chemical pathways depend on catalyst selectivity, reactor configuration, and energy intensity. Biological routes emphasize enzymatic efficiency, polymer accessibility, and hybrid integration for monomer recovery. These indicators collectively support the classification and prioritization of processing routes within circular-economy decision frameworks.

## 4. Upcycling Pathways for High-Value Recycled Polymer Materials

Upcycling emerged as a counterproposal to traditional recycling practices and was widely introduced by Michael Braungart and William McDonough in Cradle to Cradle: Remaking the Way We Make Things [69,70], where they proposed transforming discarded materials into products of higher added value through creative, ecologically oriented design [15]. Unlike conventional recycling—where materials degrade progressively, and experience reduced mechanical or economic performance after successive cycles—upcycling introduces the opposite logic: transforming waste into products with superior functional, aesthetic, or economic attributes [71].

This paradigm aligns with the principles of the CE by integrating design strategies that anticipate multiple life cycles, minimize waste from the outset, and encourage modularity and reusability. As a result, upcycling reduces energy consumption, prevents premature disposal, enables the creation of unique high-quality products, and fosters innovation from the design stage [72].

When applied to plastic waste, upcycling offers pathways to recover high-value molecules, functional materials, and specialized products that often outperform conventional polymer resins. While traditional recycling—particularly for post-consumer plastics—faces challenges such as contamination, structural degradation, and water-intensive cleaning stages [73], upcycling leverages chemical, thermal, electrochemical, biological, or hybrid conversion routes to transform carbon-rich residues into valuable resources rather than simply diverting them from landfill.

### 4.1. Technical Pathways for Plastic Upcycling

#### 4.1.1. Chemical Routes

These involve depolymerization or selective bond cleavage, including glycolysis, solvolysis, hydrolysis, alcoholysis, aminolysis, oxidation, and hydrogenolysis [41]. Polyester-based plastics (e.g., PET, PLA) exhibit high selectivity toward monomers such as terephthalic acid, dimethyl terephthalate, or ethylene glycol [74].

#### 4.1.2. Thermal and Thermochemical Process

Research on pyrolysis and gasification of plastics has emerged as a critical area of inquiry due to the escalating environmental challenges posed by plastic waste accumulation and the urgent need for a sustainable energy recovery method. Pyrolysis, which involves the thermal decomposition of plastics in the absence of oxygen to produce high-purity fuels and monomers, is a practical approach [75]. Since the early 2000s, thermochemical conversion technologies have evolved from conventional pyrolysis to advanced catalytic and microwave-assisted processes, enhancing energy efficiency and product selectivity. This versatile method converts plastics into gas by partial oxidation at temperatures above 800 °C, producing fuels or chemicals [64,76]. Despite advances, the efficient valorization of plastic waste through pyrolysis and gasification remains challenging, particularly in optimizing catalysts, reducing energy consumption, and ensuring product quality [77].

#### 4.1.3. Electrochemical Upgrading

Research on the electrochemical upgrading of PET and PLA derivatives has emerged as a critical area of inquiry, with the potential to address plastic pollution while producing valuable chemicals and green hydrogen. The field has evolved from initial studies on PET hydrolysate oxidation to advanced catalyst designs enabling industrial-scale current densities and high selectivity for products such as formate, glycolate, and hydrogen [78,79,80]. Despite progress, challenges remain in achieving efficient, selective, and economically viable electrochemical conversion of PET and PLA derivatives. Key knowledge gaps include developing cost-effective catalysts with high activity and stability at industrially relevant current densities, understanding the mechanistic pathways of selective oxidation, and optimizing process efficiency for simultaneous hydrogen production [79,81].

#### 4.1.4. Biological Upcycling

Research on the enzymatic degradation of plastics by cutinases, lipases, and carboxylesterases has emerged as a key subject of investigation due to the escalating environmental impact of plastic waste accumulation and the limitations of conventional recycling methods [82,83]. Since the 1990s, advances in understanding microbial and enzymatic degradation mechanisms have evolved, with early studies focusing on natural polymer degradation and recent efforts targeting synthetic polymers such as PET and poly(butylene adipate-co-terephthalate) [83,84].

Despite progress, enzymatic degradation of plastics faces critical challenges, including low catalytic efficiency on highly crystalline polymers, enzyme instability under industrial conditions, and limited understanding of the roles of microbial consortia in plastic upcycling. While cutinases and lipases have demonstrated activity against various polyesters, their performance on persistent plastics such as polyethylene and polypropylene remains [82,83,84,85].

#### 4.1.5. Polymer Blending and Compatibilization

Mixed plastic waste typically includes a variety of polymers, such as PE, PP, PS, and PET. These materials are often recycled from uncleaned, unsorted waste, leading to a heterogeneous mixture that complicates recycling efforts. The inherent incompatibility among these polymers results in poor mechanical properties and phase segregation when they are blended without a compatibilizer [57]; the introduction of compatibilizers, such as organic esters, acids, or modified polymers, is needed to improve the mechanical properties [10,85,86,87]. Plastic waste can be utilized in construction materials, such as concrete mixtures and block manufacturing, to produce durable, heat-resistant, and water-resistant products. The combination of plastic waste with solid waste materials such as furnace slag and fly ash can produce composite building materials that are non-toxic, odorless, and recyclable, aligning with the clean production principle [88].

### 4.2. AI as an Optimization Driver in Upcycling

Due to the high variability of plastic waste, process uncertainties, and nonlinear conversion mechanisms, upcycling systems benefit significantly from AI-enabled optimization, Table 2. ML supports predictive modeling, material discovery, and reactor-level control, helping overcome processing inefficiencies. Means et al. [86] conducted a large-scale data mining analysis of more than 10,000 published works, using natural-language processing to classify technological evolution trends. The analysis revealed that polyethylene and polypropylene are the most studied targets, that pyrolysis-based valorization is the dominant route, and that ML model integration is increasing for prediction and optimization.

Li et al. [91] analyzed the impact of the COVID-19 pandemic on the proliferation of medical plastic waste. They proposed four axes for the upcycling of plastic waste: (a) increase the biodegradability of materials; (b) transform plastics into high-value products through chemical processes; (c) promote closed recycling with biodegradation; (d) link renewable energies.

It was highlighted that AI can be integrated into catalyst design and the optimization of chemical processes, especially for predicting reaction selectivity and efficiency [92]. However, these are conceptual proposals rather than experimental applications.

The upcycling of recycled plastics, combined with residual biomass derived from agro-industrial waste, enables the production of sustainable polymers and compounds with mechanical properties comparable to, and in some cases superior to, those of virgin materials. For them, it is necessary to use AI tools that allow the optimization of 3D printing parameters, as well as the incorporation of additives—compatibilizers, stabilizers, surface modifiers, etc.—to improve mechanical strength, thermal stability, and adhesiveness, while reducing dependence on virgin materials; opening new possibilities for the creation of innovative and sustainable materials [93].

Qian and Ren [94] reviewed thermal and thermochemical upcycling technologies, including incineration, gasification, and pyrolysis, and complemented them with process optimization methodologies. The authors highlight the use of tools such as experimental design, superstructure optimization, and green supply chain management, emphasizing that AI tools and computational models, such as neural networks and vector support machines, are essential for evaluating and optimizing conversion routes of plastics into high-value products. However, they are still in stages of conceptual integration.

Khairul Anuar et al. [95] reviewed advances in recycling and upcycling of hazardous plastics, including mechanical, chemical, and biological methods. These authors highlight innovations such as enzymatic depolymerization of PET, conversion of plastic waste into carbon nanomaterials (graphene and nanotubes), and photocatalytic processes [92]. Although they recognize limitations in scalability and economic viability, they point out that AI and ML are emerging as support tools for waste classification and the optimization of chemical processes, opening the way to overcome technical and regulatory barriers in the sustainable management of plastics.

In summary, AI tools can be used to optimize, predict, and design chemical, photocatalytic, biological, and simulation processes for the upcycling of plastics, enabling the recovery of high-value-added materials and the development of new materials with novel properties in the transition towards a CE of plastics.

### 4.3. Emerging Technological and Research Trajectories

Emerging technological trajectories in plastic upcycling increasingly position AI as an enabling tool for optimizing system performance and scaling industrial deployment. AI contributes to multi-objective optimization—considering yield, purity, carbon conversion, and selectivity—while improving reactor control, predictive maintenance, and data-driven decision-making. In parallel, advanced computational tools, such as protein language models, are accelerating bio-pathway discovery, particularly in enzyme identification and metabolic engineering for polymer depolymerization [96]. Hybrid simulations combining computational fluid dynamics and ML frameworks further support process intensification under realistic industrial constraints.

Beyond purely technological advances, research is shifting toward integrating upcycled plastics with agro-industrial byproducts to produce composite biopolymers with superior properties. This direction is reinforced through AI-enabled optimization of 3D-printing parameters, simulation-driven additive selection, automated prediction of stabilizer and compatibilizer performance, and mechanochemical modeling to estimate long-term durability. As emphasized by Qian & Ren [94] and Khairul Anuar et al. [95], AI-guided optimization remains fundamental for scaling both thermochemical and biological upcycling systems to viable industrial levels, particularly in contexts where techno-economic viability, process reproducibility, and regulatory requirements still present significant challenges.

Upcycling provides value-positive material circularity by transforming post-consumer plastics into monomers, carbon-rich nanomaterials, high-performance composites, and functional biochemicals. AI-enabled strategies accelerate process efficiency, enhance selectivity, and guide new-material discovery, creating a technological pathway toward viable, scalable, high-value circularity.

This combination of design thinking, catalytic innovation, hybrid conversion routes, and AI-enabled decision systems positions upcycling as a cornerstone for the future of circular plastic systems.

### 4.4. Waste Biomaterials and Biochar as Functional Co-Feedstocks in Plastic Upcycling

Upcycling strategies can be strengthened by incorporating waste biomaterials—particularly plant residues (e.g., bagasse, rice husk, wheat straw, corn stover, fruit/vegetable pomaces) and animal-derived wastes (e.g., poultry feathers/keratin, crustacean shells as chitin/chitosan precursors, eggshell-derived CaCO_3_, and manure-derived solids)—as low-cost, regionally available co-feedstocks [65]. In practice, these streams can be used as (i) lignocellulosic fibers/flours to reinforce recycled polyolefins and PET, (ii) bio-derived functional additives (e.g., chitosan-based phases), or (iii) mineral/biogenic fillers (e.g., eggshell CaCO_3_) that improve stiffness and dimensional stability in non-critical components. A particularly promising derivative is biochar, obtained via pyrolysis/thermoconversion of plant or animal wastes, which provides a carbon-rich, porous, and thermally stable filler. When properly milled and dispersed, biochar can contribute to mechanical reinforcement, UV/thermal stabilization, and (in some formulations) improved barrier or electrical properties, enabling upgraded products such as construction profiles/panels, decking, pallets/crates, and other durable applications [97,98].

However, waste-biomaterial-based upcycling requires explicit control of variability, moisture affinity, interfacial adhesion, and contaminant profiles. Effective implementation typically involves pretreatments (drying, sieving, de-ashing), compatibilization/coupling (e.g., maleated polyolefins for PE/PP), and quality assurance to mitigate odor, leaching risks (metals/PAHs), and property drift. Accordingly, these hybrid routes should be evaluated with standardized mechanical testing and application-relevant safety/regulatory screening to ensure that performance gains are achieved without shifting burdens downstream [99,100,101].

### 4.5. Feasibility Gates for Upcycling: Thermodynamics/Energy, Catalysts, and Techno-Economics

Upcycling is often presented as a route to increase circular value beyond conventional recycling; however, its feasibility depends on three coupled “gates” that must be satisfied in practice: (i) thermodynamics/energy; (ii) catalysts and feedstock tolerance; (iii) techno-economics at scale.

Energy and thermodynamic constraints (Gate 1) are central for thermochemical upcycling (e.g., catalytic pyrolysis, hydrocracking, gasification), where high temperatures and heat duty can dominate the overall environmental and economic performance. Feasible deployment, therefore, relies on heat integration, energy recovery, and targeting products toward higher-value slates (chemicals/monomers) rather than fuel-only outputs, particularly when feedstocks are heterogeneous.

Catalyst constraints (Gate 2) frequently determine operational robustness. Real waste plastics contain additives, fillers, pigments, and halogenated fractions that can promote coking, poison active sites, and shift selectivity. Hence, catalyst design must be assessed alongside deactivation/regeneration behavior, tolerance to chlorine/metals, and the need for pre-sorting/decontamination. The sensitivity of yields and product distributions to operating conditions and blended waste inputs is illustrated by co-thermochemical processing studies [22], reinforcing that “one-size-fits-all” upcycling is rarely realistic without feedstock conditioning.

Economic constraints (Gate 3) extend beyond reactor performance: the dominant cost drivers often include collection and pre-treatment, sorting/washing, removal of problematic fractions (e.g., PVC), catalyst makeup and lifetime, CAPEX for upgrading/separation, and compliance with emissions constraints. Recent TEA/LCA studies show that profitability and climate benefits are highly scenario-dependent and are strongly affected by the value of the product slate, energy prices, and plant integration.

Taken together, these gates indicate that upcycling is most feasible when (a) feedstock quality is controlled (via advanced sensing/sorting), (b) catalysts and separations are matched to contamination and degradation states, and (c) the targeted outputs provide sufficient value to justify the additional energy and processing requirements. This feasibility framing also clarifies why AI-enabled sensing and route selection are critical enablers for implementing upcycling under real-world constraints.

## 5. Life Cycle Assessment and Environmental Metrics in Circular Plastic Systems

Life Cycle Assessment (LCA) has emerged as a fundamental analytical tool for evaluating the environmental burdens associated with plastics throughout their life cycles. Its methodological foundations evolved from early Resource and Environmental Profile Analyses (REPA) during the 1960s–70s [102], originally applied to compare beverage packaging alternatives, leading eventually to the standardized framework established during the 1990s [103,104], defining requirements for goal definition, functional units, system boundaries, inventory compilation, and impact interpretation [103,104,105]. These standards formalized requirements for system boundaries, functional units, life-cycle inventory development, and impact interpretation, enabling methodological transparency across material systems.

In the context of plastics, LCA has gained prominence due to increased attention to emissions from petrochemical-origin polymers, landfill persistence, and microplastic generation [103]. Mechanical recycling often yields favorable environmental outcomes and lower global warming potential (GWP) than virgin polymer production. However, its effectiveness is constrained by feedstock heterogeneity, accumulated degradation, loss of mechanical performance, and contamination effects [104]. In contrast, chemical recycling—especially pyrolysis and depolymerization pathways—enables monomer-level recovery and higher substitution equivalence but typically exhibits greater energy demands and process-related emissions, which vary significantly depending on reactor configuration and energy source [105]. Emerging enzymatic and biological depolymerization pathways provide monomers of high purity, although current energy limitations and uncertain industrial scalability introduce environmental trade-offs [106]. Upcycling systems, exceptionally when engineered for enhanced performance and extended product lifetime, can yield environmental gains, provided that substituted virgin materials are explicitly accounted for [107].

### 5.1. System Boundaries Relevant to Plastic Circularity

The environmental interpretation of recovery routes depends heavily on the selection of system boundaries. The classification most commonly applied in plastics LCA literature includes Cradle-to-Gate, Gate-to-Gate, Cradle-to-Grave, and Cradle-to-Cradle boundaries [108]. These boundaries determine whether environmental burdens will be attributed only to conversion stages or also to storage, use phase, and subsequent end-of-life pathways.

For complex circular scenarios, boundary selection must explicitly represent (i) multi-cycle loops with progressive property loss (“spirality”); (ii) open-loop cascade/downcycling where the recycled material serves a different function than the virgin benchmark; (iii) cross-regional transport and processing, which can dominate impacts through logistics and regional energy mixes. Multi-cycle modeling should state the number of loops considered, the quality-retention assumption (e.g., a quality factor or property-based discount), and the allocation/substitution rule used to credit displaced virgin production. For open-loop/downcycling, the functional unit should reflect the new application and the substitution ratio should be justified (often <1:1). For cross-regional systems, boundaries should explicitly include collection, baling, shipping, sorting, and reprocessing distances and should regionalize electricity/heat and end-of-life practices to avoid misleading comparisons across geographies.

A comparative summary is presented in Table 3 to facilitate alignment among the recovery process type, the intended system scope, and the methodological rationale.

### 5.2. Performance Characterization of Chemical Recycling Routes

Chemical valorization routes—such as catalytic pyrolysis, glycolysis, methanolysis, alcoholysis, and oxidative fragmentation—restore polymer-derived carbon into usable precursor molecules. LCA outcomes demonstrate that the environmental benefits of chemical recycling depend strongly on conversion yield, energy source, solvent recirculation rates, catalyst life, and product recovery efficiency [105]. Under fossil-derived heating, thermochemical conversion may exceed the energy demand of mechanical recycling; however, when powered by electrified low-carbon energy systems, monomer-grade outputs exceed the virgin displacement threshold and yield net GWP reductions [105,106].

### 5.3. Life-Cycle Implications of Upcycling Processes

Upcycling enables the recovery of value-enhanced outputs, such as nanocomposites, compatibilized blends, high-performance carbonaceous materials, and additive-modified formulations, yielding products that outperform the original resins [107]. Unlike classical recycling, which typically reduces mechanical performance and polymer chain integrity over time, upcycling extends the life cycle and delays disposal. LCA comparisons show that when performance equivalence with high-grade virgin resin is achieved, avoided burdens outweigh additional material additions; however, the evaluation must quantify emissions associated with compatibilizers, catalyst synthesis, and dispersive processing.

### 5.4. Digital-Twin-Based LCAs and Computational Attribution

Recent studies incorporate digital twin models in LCA frameworks to simulate reactor thermodynamics, conversion yield distributions, degradation kinetics, and catalyst regeneration cycles [105]. These models reduce allocation uncertainty by incorporating real-time process data. Simulation-assisted LCAs have quantified up to 25% variation in unit energy impacts depending on residence time and waste-composition fluctuation scenarios, strengthening scenario-based environmental predictions linked to industrial adoption readiness.

### 5.5. Comparative Findings Across Recycling Pathways

Cross-method assessment indicates that no individual technological pathway offers uniform environmental superiority. Mechanical recycling has the lowest energy intensity but suffers from quality attrition when input contamination exceeds thresholds [104]. Chemical recycling achieves the highest monomer purity and closed-loop value recovery but involves energy-intensive stages whose net benefit materializes only under adequate purification and solvent recovery conditions [105]. Biological depolymerization offers maximal theoretical circularity by producing monomers equivalent to virgin stock with minimal toxins [106]; however, maturity and processing yield limitations remain. Upcycling, when linked to demonstrable substitution of engineering-grade resins, appears promising in long-horizon LCA scenarios [107]. Instead, systemic decisions must be informed by environmental metrics derived from robust LCA approaches. Across technologies, environmental trade-offs arise from differences in energy demand, process intensification, additive impacts, and the substitutability of recycled materials. For instance, although mechanical recycling provides significant reductions in energy consumption and greenhouse gas emissions relative to virgin resin [104], its benefits diminish rapidly when feedstock heterogeneity, contamination, or polymer degradation degrades material quality. Conversely, chemical routes—particularly pyrolysis and depolymerization—enable high-purity outputs and valorization of complex waste streams, but they involve thermal hotspots that increase energy burden and emissions unless improved energy integration and catalytic optimization are implemented [105]. Recent studies report that closed-loop depolymerization routes can match virgin performance from a functional perspective; however, their environmental profiles depend on the energy sources and conversion efficiencies used [60,61].

Bioconversion-based routes have emerged as highly promising pathways, particularly for PET, where enzymatic depolymerization produces monomers with minimal compositional variability. In LCA scenarios, biocatalytic depolymerization shows potential to outperform chemical pyrolysis due to lower thermal requirements and higher molecular circularity, provided that conversion rates, retention yields, and enzyme stability are optimized [113]. Nonetheless, uncertainty persists, as large-scale enzyme production introduces environmental burdens associated with nutrient media, purification, and temperature control—requirements often overlooked in conventional impact models [114]. In this context, policy frameworks must facilitate industrial deployment by accounting for upstream burdens and incentivizing stable energy integration, renewable-based heating, and local valorization cycles.

Upcycling represents a differentiated scenario. While material quality frequently surpasses that of standard recyclate, environmental burdens depend on additives, nanofillers, compatibilizers, and stabilizers, whose production may have non-negligible impacts [107]. Studies integrating consequential LCA trends indicate that upcycling is justified when it demonstrably prevents production of energy-intensive virgin substitutes, particularly in packaging, electronics, engineered parts, and hybrid biodegradable composites [89,115]. Under such conditions, substitutability factors improve significantly, allowing circular materials to displace virgin grades at ratios of 0.8–1.0 based on mechanical properties, processability, and durability [116]. This makes upcycling strategically relevant for countries with limited high-quality sorting infrastructure, since value addition compensates for feedstock limitations.

From a policy standpoint, regulatory instruments must integrate environmental metrics, technological maturity, and evidence of substitutability. EPR schemes have demonstrated measurable improvements in recovery rates when linked to material quality requirements and LCA-based incentives [108]. However, EPR remains insufficient without digital traceability that can attribute environmental burdens to specific waste sources. Recent frameworks incorporating blockchain-based material passports and AI-based sorting quality indices indicate that traceability correlates with improved recyclate consistency, reduced rejection fractions, and decreased transport-induced emissions [89].

International guidelines also recommend adopting harmonized functional units that reflect real service performance rather than simple mass equivalence (ISO 14040; ISO 14044) [103,104]. Studies comparing one kg-gate outputs of recycled resin display bias when downstream performance differs substantially, especially under mechanical degradation or quality downgrading. Alternative functional units—such as equivalent protection time in packaging, mechanical strength-adjusted kilogram equivalents, or normalized service lifetime—yield clearer environmental profiles [117]. New methodological work on circularity metrics advocates integrating substitutability factors (αrec:vir), material retention coefficients, and cumulative energy demand to identify optimal technology deployment scenarios.

In addition to policy and methodological standardization, institutional stability is essential. Countries that have achieved consistent circularity gains—including Germany, The Netherlands, and Japan—share three structural elements:

- Long-term regulatory planning;
- Industrial-academic knowledge networks that reduce technological uncertainty;
- Subsidies or tax relief for closed-loop reintegration, particularly for sectors requiring technical-grade recycled feedstocks [118,119].

Implementing these mechanisms in Latin American contexts requires adaptation to regional socioeconomic conditions, informal waste systems, and infrastructure constraints. Finally, integrating AI enhances environmental decision-making through multiple mechanisms. ML-based predictive allocation models reduce sorting losses, increase material purity, and support dynamic LCA scenarios. Surrogate-based energy predictions currently allow estimating reactor-level sensitivity (~20–40% reductions reported in pyrolysis optimization scenarios), while digital twins enable anticipatory maintenance, reduced batch rejection, and quality-tracking feedback loops. Such improvements demonstrate measurable reductions in environmental hotspots across recycling and upcycling chains [58,120].

In synthesis, achieving environmentally robust circular plastic systems requires: (i) coupling LCA-based decision frameworks with technology-specific operational data; (ii) promoting substitutability-validated outputs rather than generalized recycling rates; (iii) systematically applying AI for efficiency gains and uncertainty reduction; (iv) integrating policies that internalize environmental externalities rather than shifting them between stages.

With these combined strategies, circularity transitions become not only technologically plausible but also socio-environmentally resilient, widening opportunities for carbon mitigation, waste reduction, and regenerative material flows.

## 6. Policy Instruments, Institutional Frameworks, and Circular Governance Mechanisms

Circular systems for plastics require not only technological innovation and advanced recycling pathways, but also robust institutional architectures that enable accountability, traceability, and economic viability. Among these instruments, EPR has emerged as a central regulatory pillar, complemented by market incentives, infrastructure development strategies, and supply-chain alignment policies. These tools aim to internalize the environmental costs of post-consumer plastic flows and to foster investment in durable, circular systems [121,122]. Below, we summarize the key operational and governance mechanisms associated with circularity, emphasizing their structure, purpose, and implementation challenges.

### 6.1. Extended Producer Responsibility

EPR is a regulatory approach that reallocates the burden of waste management to those who introduce products to the market, rather than to municipalities or end consumers. This principle ensures that producers assume financial and operational responsibility throughout the life cycle of their products, facilitating better material recovery, eco-design, and transparent accountability mechanisms. Its conceptual foundations are rooted in environmental economics, particularly the principle “who pollutes pays” [112].

EPR programs typically pursue four strategic goals: (i) waste prevention by extending product durability and encouraging reparability; (ii) redesign based on recyclability, modularity, and reduced toxicity of components; (iii) increased material recovery through funded collection systems, reverse logistics, and valorization chains; (iv) internalization of environmental externalities into business models. These objectives are operationalized through instruments such as organized collection systems, eco-contributions, selective return programs, deposit-refund schemes, and voluntary or mandatory take-back programs [122].

### 6.2. Regulatory Framework for EPR Implementation

Formal adoption of EPR requires legal definitions for producers, categories of regulated products, financial obligations, traceability tools, and sanction structures. Regulatory frameworks typically define: (i) what constitutes a “producer”; (ii) target product categories (e.g., packaging, electronics, tires, batteries); (iii) collection and recycling quotas; (iv) reporting obligations and traceability systems; (v) penalty schemes in cases of non-compliance

This framework ensures institutional enforceability and prevents competitive asymmetries. Existing Latin American frameworks still exhibit fragmentation; in Mexico, regulatory implementation remains limited due to weak sanction systems, but recently, a law on EPR and bioeconomy was announced by deputies [123,124].

Recent federal initiatives propose the strengthening of EPR within the legal architecture of a national CE policy, including fiscal incentives, mandatory recovery targets for specified products, harmonized registries, and institutional coordination among municipalities, states, and federal agencies.

### 6.3. Refuse-Derived Fuel and Energy Valorization Pathways

Refuse-Derived Fuel (RDF) has gained prominence as a strategy for energy recovery from mixed municipal waste streams, serving as an alternative when mechanical or chemical recycling is not viable. RDF is generated through a sequence of separation, size reduction, drying, and purification stages, yielding a combustible fraction suitable for industrial kilns and co-combustion systems [125,126]. A central benefit of RDF is its capacity to displace conventional fossil fuels—particularly coal, diesel, and natural gas—in high-temperature industrial processes, thereby reducing direct process emissions and facilitating lower-carbon energy matrices [127]. Its use in cement kilns and power plants has demonstrated measurable rates of fossil fuel substitution, aligning with decarbonization objectives in energy-intensive sectors [128,129].

From an operational perspective, RDF valorizes non-recyclable fractions—such as composite plastics, laminated papers, textiles, and contaminated post-consumer residues—through mechanical refinement and thermochemical stabilization [130,131]. This redirection of non-recoverable waste into energy streams reduces environmental burdens associated with unmanaged disposal. It mitigates long-term impacts, such as methane emissions, leachate generation, and land occupation, in landfill facilities [127,132]. Additionally, RDF production supports integrated waste management frameworks by prioritizing material recovery, segregation, and recycling of high-value fractions while reserving residual material for energetic valorization [129].

Crucially, pre-treatment operations confer greater uniformity in physico-chemical characteristics, resulting in fuel with predictable calorific values, moisture content, and ash composition [133]. This stabilization enhances combustion stability and facilitates its integration into industrial systems designed for coal-based inputs. Standardization initiatives—including classification guidelines developed by entities such as the National Centre for Resource Recovery—aim to establish accepted performance thresholds that foster wider market acceptance and cross-plant interchangeability [130].

Despite these benefits, widespread RDF deployment faces persistent challenges, including feedstock heterogeneity, emission control during combustion, public perception issues, and regulatory limitations in some jurisdictions. The environmental performance of RDF systems depends strongly on the consistency of pre-treatment, the co-combustion technology, and the pollutant mitigation infrastructure. Consequently, ongoing technological improvement, targeted regulatory incentives, and long-term industrial planning remain necessary to increase RDF uptake and fully integrate it as a complementary pathway within circular waste-to-resource frameworks [127,128].

From an industrial CE perspective, coupling RDF/thermochemical valorization with AI-enabled monitoring and decision-support can help move integrated facilities toward near-zero-waste operation by dynamically allocating each fraction to the highest feasible value pathway under local constraints (quality, emissions, economics, and infrastructure).

### 6.4. Municipal Solid Waste (MSW) Systems in Circular Transitions

MSW is the primary input stream from which recyclable fractions of commercial value are extracted. Its composition varies depending on socioeconomic development, urbanization, climate, and consumption patterns. However, typical profiles report between 40–60% organic residues, approximately 10–15% plastics, and smaller proportions of metals, glass, textiles, and inert fractions [134,135,136]. When MSW is not managed adequately, its accumulation generates methane emissions, promotes soil and groundwater contamination, and increases public expenditure due to landfill expansion and transport logistics [137,138,139].

Importantly, these city- and region-specific conditions are tightly coupled to human lifestyles: consumption patterns, housing density, and household participation in source separation directly shape MSW composition, contamination levels, and capture rates. Consequently, the urban context should be treated as a primary driver of circular performance, rather than a secondary boundary condition.

Circular transitions in USW governance have shifted away from solely end-of-pipe disposal strategies toward integrated systems that begin at the household and commercial source. Contemporary systems promote mandatory source separation to increase recovery efficiency, while organic fractions are increasingly managed through composting and anaerobic digestion programs, reducing landfill loads while generating biofertilizers or biogas. Recyclable flows are routed to classification centers that increasingly use optical sensors and AI-driven sorting algorithms to differentiate polymer grades, colors, contaminants, and degradation states in real time. Mixed residual streams are treated through controlled energy-valorization schemes, particularly in regions lacking infrastructure for extensive mechanical recovery. Additionally, financial instruments such as pay-per-generation tariffs are being implemented to internalize environmental costs and incentivize behavioral change.

Current international guidelines emphasize that the evolution of USW systems must be evaluated not only by diversion rates but also by environmental–economic performance metrics, including emission reductions, avoided land occupation, and the efficiency of material recirculation [140,141,142]. In this context, aligning USW systems with circularity principles transforms waste from a municipal liability into a strategic asset that feeds industrial recovery chains, supports localized job creation, and enables measurable sustainability gains [140,143].

### 6.5. Materials Recovery Facilities (MRFs)

Materials Recovery Facilities (MRFs) constitute the operational backbone of circular plastic systems because they separate recyclable fractions at an industrial scale and ensure that materials reach downstream recovery processes under acceptable quality conditions. These facilities operate under two predominant modalities: those receiving pre-segregated recyclable flows, often referred to as clean MRFs, and those that process unsorted municipal solid waste through intensive sorting operations, commonly known as dirty MRFs. Independent of the modality, MRFs integrate sequential processing stages that may include magnetic separation for ferrous metals, optical and spectroscopic classification to differentiate polymer types, pneumatic systems for density-based fractionation, and quality-assurance lines where manual or automated inspection removes contaminants before bale consolidation [35,144].

Optimizing MRF performance directly supports reductions in landfill volumes, increases in recyclate purity, and improvements in the commercial value of post-consumer secondary materials. Beyond environmental benefits, improved MRF throughput contributes to regional economic activity by creating jobs and stabilizing supply chains for recycled resin. Recent assessments emphasize that financial feasibility is highly dependent on scalability and geographical context; in urban or metropolitan areas with high material density, economies of scale enable recovery efficiencies and revenue levels unattainable in smaller, more dispersed territories [145,146]. Thus, MRFs not only serve as sorting infrastructures but also as platforms that influence the quality, flow consistency, and long-term viability of circular plastic markets.

### 6.6. Supply Chain Management (SCM) for Circular Materials

Circular supply chains encompass the coordinated return of materials, quality-assured feedstock flows, and dynamic logistical planning to reduce losses and stabilize secondary-material markets. In modern systems, supply-chain operations are increasingly mediated by digital infrastructures that track material performance across the entire reverse logistics pathway. This includes integrating IoT-enabled monitoring for real-time assessment of processing conditions, enterprise-level ERP platforms that synchronize procurement and post-consumer feedstock allocation, and blockchain-based traceability modules that secure chain-of-custody information and compliance records. Additionally, advanced predictive analytics allow forecasting of demand, availability, and degradation risk, enabling allocation of recycled resin to suitable applications before quality loss occurs. When combined with reverse logistics networks that efficiently return materials from distributed collection points, these tools improve forecasting accuracy, optimize transportation scheduling, reduce losses from contamination or storage delays, and facilitate near-real-time environmental accounting. SCM integration has proven fundamental to increasing competitiveness in recycling-dependent industries [147,148,149].

The instruments described above are not isolated components; instead, they operate synergistically to support the evolution towards high-performance circular systems. EPR incentives drive eco-design; MSW and MRF infrastructures provide the practical material streams for recovery; RDF strategies ensure energetic valorization of non-recoverable mixed fractions; and SCM frameworks enable reliable flow management, value retention, and economic integration.

When deployed consistently within long-term institutional planning horizons, these policies and industrial mechanisms promote structural circularity, reduce environmental burdens, and increase the substitution of secondary materials for virgin feedstocks.

## 7. Case Studies, Future Directions, and Framework for Circular Plastic Systems

Addressing the global plastic crisis requires systemic evolution from linear take–make–dispose models toward circular configurations that preserve material value through design, reuse, and technologically enabled recovery pathways. CE frameworks provide the methodological structure to close resource loops, reduce environmental burdens, and generate economic value from previously discarded assets [150]. The following case studies are presented comparatively to highlight how regional governance, infrastructure maturity, and lifestyle-linked participation patterns shape feasible circular pathways and measurable outcomes. The evidence increasingly shows that circularity performance depends not only on technological optimization but also on institutional stability, traceability infrastructures, and economic incentives that reinforce recycling behaviors [110,151]

### 7.1. Global Case Studies: Insights and Quantitative Evidence

The transition toward circular plastics has progressed unevenly across regions, but a growing body of international experiences offers valuable lessons for technical viability, policy design, and system integration. The following case studies highlight approaches undertaken across different geographies, revealing trends in technology adoption, governance structures, and the role of economic and social factors in implementing CE strategies for plastics.

#### 7.1.1. Middle Eastern Industrial Circularity: SABIC and Aramco Initiatives

In Saudi Arabia, SABIC and Aramco have consolidated industrial-scale initiatives focused on polymer depolymerization, catalytic pyrolysis, polymer-to-polymer recovery, and monomer purification [152]. Pilot-scale analyses indicate that pyrolysis oils and recovered olefinic streams attain quality comparable to that of virgin feedstocks when integrated with post-processing purification [153]. LCA estimates report that process impacts are highly dependent on the energy mix; when low-carbon energy is introduced, net cradle-to-gate GWP reductions of 12–22% relative to virgin resin production are achieved [152,154]. The lesson from these programs highlights that the success of circularity at an industrial scale requires: (i) digitalization for material traceability; (ii) unified standards for recycled polymer quality; (iii) synergistic interactions between private and public shareholders [152]. They also underscore the challenge of balancing the energy-intensive chemical recycling process with climate mitigation goals, emphasizing the need to decarbonize energy sources.

#### 7.1.2. Thailand’s Rayong Multi-Stakeholder System: The Central Role of Informality

The Rayong province in Thailand offers a contrasting case, emphasizing the importance of socioeconomic factors. Here, a multi-actor recycling system comprising households, community groups, informal collectors, and small sorting centers achieves material recovery rates ranging from 11.3% to 64.1%, depending on the waste stream and participation levels [155]. The Thai experience shows that recognizing and supporting informal collections networks can enhance income generation for workers in low- and middle-income settings [156]. However, the observed variability also reveals vulnerabilities, including inconsistent source separation, insufficient enforcement of waste management regulations, and market volatility in recyclable materials. The Rayong case underscores that technology alone cannot achieve circularity; it must be accompanied by equitable governance, long-term social integration, and economic incentives [155,157].

#### 7.1.3. Malaysia’s P-Graph–Integrated EPR Evaluation for Circular Plastics

Malaysia’s integration of mathematical optimization into EPR evaluation provides a notable example of how circular policy outcomes can shift when full external costs are incorporated. Using the P-graph methodology, Malaysian analysts quantified the environmental and economic consequences of different end-of-life pathways, revealing that mechanical recycling produces a positive externality of approximately +12.10 USD per ton. In comparison, incineration generates a negative externality of around −199.58 USD per ton [158]. These results illustrate that strategies that appear favorable when evaluated solely on direct operational costs may, in fact, impose broader socio-environmental burdens.

The P-graph framework contributes uniquely to this assessment by formally representing the complex interactions among producers, recyclers, logistics operators, and policy agents. Its capability to map optimal pathways in multi-actor networks strengthens EPR schemes by clarifying how economic and environmental responsibilities should be distributed across the system [158]. Beyond quantifying externalities, the methodology improves decision-making by distinguishing pathways that genuinely add net value from those whose performance depends on unpriced external costs.

Broader implications of this approach extend beyond Malaysia. Evidence from comparative studies suggests that variability in EPR performance often emerges from differences in how responsibility is allocated, particularly when financial responsibility systems are not aligned with physical responsibility outcomes [159]. Likewise, the effectiveness of EPR schemes is influenced by producer-level incentives—where individual-based schemes generally outperform collective systems due to more transparent accountability for product design and end-of-life returns [121,122,160]. Taken together, these findings reinforce the need for circular transition pathways to account not only for direct processing costs but also for externalities, governance structures, and incentive mechanisms that determine system durability and economic fairness.

#### 7.1.4. The PHOENIX Framework in the European Union: Design for Plasmix Circularity

Within the European Union, the PHOENIX project introduced a comprehensive framework for addressing the circularity of plasmix, a heterogeneous fraction of post-consumer plastics traditionally considered unsuitable for high-value recycling [160]. This framework integrates product design principles, market-acceptance criteria, and multi-criteria decision-making tools to evaluate circular solutions. PHOENIX highlights the potential of design for recycling and of design-from-recycling strategies to enable the valorization of mixed plastic waste streams. Its relevance to global circularity lies in demonstrating that systemic design, rather than material purity alone, can unlock new pathways for circular markets, particularly for regions with high waste heterogeneity [160,161,162]. However, challenges remain, such as the need for improved sorting technologies and the development of markets for recycled products, which are critical for achieving higher recycling rates and closing the plastic loop [51].

#### 7.1.5. PET Circularity Through LCA and MFA Integration

A global comparative study on PET circularity illustrates how integrating LCA and MFA can reveal trade-offs among different recycling technologies. Enzymatic depolymerization consumes approximately 57 MJ/kg PET, while methanolysis requires 38 MJ/kg PET, with associated greenhouse gas emissions of ~3.0 and ~2.0 kg CO_2_e/kg, respectively. Hybrid scenarios combining mechanical and chemical recycling show the potential to reduce virgin PET demand by 56% and waste generation by 64%. These results highlight that chemical pathways can complement mechanical recycling but must be evaluated under energy decarbonization scenarios to ensure climate benefits [21,59,76,116,163].

#### 7.1.6. Cameroon’s Emerging Circularity Efforts

In Cameroon, longitudinal studies show that the circular plastics sector is still in an embryonic stage, constrained by limited institutional capacity, insufficient infrastructure, and underdeveloped markets. Nonetheless, pilot initiatives involving community sorting programs and small-scale recycling cooperatives reflect early opportunities to build bottom-up models. Lessons from Cameroon warn that without foundational policy alignment, data systems, and supportive governance structures, circularity efforts risk stagnation [164,165,166]. While the circular plastics sector in Cameroon faces significant challenges, the potential for community-driven initiatives to contribute to a circular economy is evident. However, without the necessary policy support and infrastructure development, these efforts may not reach their full potential.

### 7.2. Latin America Case Studies: Policy, Socioeconomic Context, and Circularity Pathways

Latin America exhibits diverse trajectories in adopting circular plastic practices. While several countries have advanced regulatory frameworks or industry initiatives, the region also faces systemic challenges, including informality, infrastructure limitations, and inconsistent enforcement.

#### 7.2.1. México

Mexico has one of the highest PET recovery rates in the region, exceeding 60%, driven primarily by private-sector initiatives and a robust PET recycling industry. The informal sector—estimated at over 35,000 workers—plays a crucial role in supplying recyclables. Despite the existence of CE-oriented guidelines, significant gaps persist in harmonizing EPR implementation and ensuring nationwide traceability systems. However, the recently enacted law on EPR and bioeconomy could reduce these gaps [124]. Mexico’s progress suggests that strong industry involvement can accelerate circularity but requires unified policy frameworks to ensure long-term system stability [156,164,166,167,168].

#### 7.2.2. SubAmerica

Brazil illustrates one of the most complex contexts for circular plastic transitions. Although national recycling rates remain near 1%, the informal sector contributes up to 90% of recovered materials, forming the backbone of local supply chains. Studies on HDPE indicate a recovery of nearly 38%. However, rejection rates approach 29% due to contamination and insufficient sorting, reinforcing the need for subsidized infrastructure, cooperative models such as PUES, and gradual formalization pathways [169,170,171]. While the informal sector plays a pivotal role in Brazil’s recycling efforts, the transition to a CE faces significant challenges. These include the need for improved infrastructure, policy support, and technological innovations to enhance material quality and reduce rejection rates.

Chile represents a contrasting scenario, being the only country in the region with a formally enacted EPR law for packaging. This milestone has accelerated efforts toward traceability, blockchain-enabled monitoring, and standardization of recyclate quality, though early implementation still depends on stronger compliance mechanisms and reliable monitoring systems [169,172]. While the EPR law in Chile represents a progressive step towards sustainable waste management, its effectiveness depends on overcoming compliance and monitoring challenges.

Colombia has advanced PET circularity through producer agreements and private-sector investment. Systems dynamics analyses show that feedback loops among collection capacity, recovery rates, and market demand determine long-term performance, highlighting the need for coordinated policy-industry alignment [158]. Despite these advancements, significant challenges persist, including Colombia’s low overall recycling rate of 17%. Such limitations underscore the urgent need to strengthen public engagement and expand infrastructure to achieve more effective waste management.

In Argentina and Peru, CE policies exist on the planning agenda, but implementation remains limited. Regulatory discontinuity, weak infrastructure, and insufficient incentives constrain the scalability of recovery systems [170].

Costa Rica shows promising environmental awareness and potential for community-based circular initiatives; however, the absence of unified material-traceability systems restricts operational deployment. Strengthened institutional frameworks will be required to translate CE discourse into measurable outcomes [170,171]. Costa Rica faces challenges in implementing CE strategies, but the potential for success is significant with the proper institutional and technological support. The integration of advanced technologies and improved regulatory frameworks can facilitate this transition, enhancing resource efficiency and sustainability.

Together, these cases demonstrate that advancing circularity relies not only on technology but on regulatory stability, infrastructure investment, and equitable inclusion—particularly of informal workers—to sustain long-term material recovery and recycling outcomes in Latin America.

Taken together, these Latin American cases illustrate how circular outcomes are co-determined by (i) collection and sorting infrastructure; (ii) the role and organization of the informal sector; (iii) policy instruments (e.g., EPR scope, enforcement, and traceability); (iv) household-level source separation that reflects local lifestyles and service access. For example, Mexico’s comparatively high PET recovery has been enabled by long-standing collection value chains and informal-sector participation, yet gaps remain in traceability and consistent upstream separation. In contrast, Brazil’s low national recycling rates are amplified by contamination and high rejection fractions, indicating that downstream capacity alone cannot compensate for weak source segregation and inconsistent collection coverage. Chile’s EPR-driven framework signals progress toward standardized obligations and traceability, but performance ultimately depends on local implementation capacity and citizen participation. These comparisons reinforce that ‘best’ pathways are context-specific and that AI-enabled sorting and routing strategies must be designed with regionally grounded constraints in mind.

### 7.3. Cross-Cutting Lesson from Global and Regional Experiences

Analysis of these case studies reveals several key patterns. Firstly, informal networks are critical in developing economies: In Latin America and Southeast Asia, informality accounts for the majority of material recovery, and excluding informal actors risks collapsing existing value chains. Secondly, external costs fundamentally alter optimal system design; Malaysia’s experience demonstrates that accounting for environmental and social impacts transforms both cost calculations and technology preferences. Thirdly, circularity does not inherently guarantee climate benefits since the energy intensity of chemical and biological recycling can exceed that of mechanical routes unless supported by low-carbon energy sources. Fourthly, data and traceability systems are foundational, as the absence of a unified data infrastructure limits effective monitoring and compliance enforcement, as well as the development of recycled polymers markets. Finally, systemic design enables effective management of material heterogeneity: frameworks such as PHOENIX demonstrate that mixed plastics (plasmix) can achieve circularity when product design, separation strategies, and market acceptance criteria are jointly integrated.

### 7.4. Future Directions for Circular Plastic Systems

Future directions for circular plastic systems emphasize the growing importance of digitalization, advanced recycling technologies, social inclusion, and standardized assessment frameworks. Progress in circularity will increasingly rely on Industry 4.0 tools—such as advanced sorting, AI-enabled sorting, blockchain-based traceability, and digital twins—which are essential for ensuring chain-of-custody compliance, verifying recycled content, and improving system-wide efficiency. At the same time, high-value chemical and biological recycling technologies, including solvolysis, glycolysis, methanolysis, and enzymatic hydrolysis, are expected to expand, particularly for PET and polyamides; however, their effective deployment depends on decarbonized energy systems and harmonized life-cycle assessments to safeguard environmental integrity. Equally critical is the advancement of socially inclusive circularity models: evidence from Latin America and Africa demonstrates that cooperatives, share-infrastructure arrangements, and micro-franchising can strengthen socioeconomic resilience, ensure fair compensation, and improve occupational safety for workers who sustain material recovery. Finally, the future of circular plastics requires standardized metrics and harmonized LCA/TEA methodologies to enable consistent comparison across technologies, as well as policy alignment—through coherent EPR regulations, market incentives, and trade measures—to secure the competitiveness of recycled polymers relative to virgin materials.

### 7.5. Toward a Coherent and Inclusive Framework for Circular Plastic Systems

Building on the case studies and analytical evidence, an integrated framework for circular plastic systems must operate across multiple levels of decision-making. At the strategic level, the ReSOLVE framework—comprising the actions Regenerate, Share, Optimize, Loop, Virtualize, and Exchange—provides a macro-level structure for planning circularity pathways and identifying leverage points for system transformation. At the design and product level, the PHOENIX framework offers guidance for addressing material heterogeneity and enabling design for recycling and design from recycling strategies, particularly for complex fractions such as plasmix. Optimization and decision support should incorporate P-graph methodologies to evaluate system-wide performance under economic, environmental, and social externalities. Environmental assessment requires harmonized integration of LCA and MFA to quantify impacts consistently and guide investment and policy prioritization. Finally, operational deployment depends on digital traceability infrastructure—including blockchain, digital tagging, and AI-enabled sorting—that ensures chain-of-custody integrity, quality control, and compliance with recycled-content standards. Together, these components form a coherent, data-driven, and socially inclusive framework capable of supporting CE transitions from national policy design to local recycling operations.

## 8. Discussion

Despite sustained global advances in technologies and governance mechanisms for circular plastics, important systemic gaps remain that constrain measurable environmental outcomes. The integration of mechanical, chemical, and biological pathways has generated diverse circular options. Nevertheless, their real environmental benefits often remain uncertain when full life-cycle performance, substitution rates, and degradation-dependent quality declines are considered [51,110,142,155]. In practice, most LCAs still rely on mass-equivalent functional units, a limitation that obscures key performance differences among recycled resin grades, particularly in packaging and engineering-level applications [117,165]. Evidence increasingly suggests that service-life-equivalent functional units, durability-normalized metrics, and barrier-performance-based evaluations yield clearer environmental signals, especially when recyclate properties deviate from virgin benchmarks [119,164].

Chemical recycling has emerged as a strategically relevant complement to mechanical systems, especially for heterogeneous streams and multilayer structures. However, upscaling remains hampered by energy demand, catalyst turnover, and carbon intensity associated with high-temperature depolymerization [60]. These inefficiencies are not merely technological; they reflect weaknesses in analytical integration, as TEA-LCA coupling rarely accounts for real-world catalyst loss, downtime, or suboptimal conversion fractions [164]. Similarly, enzymatic depolymerization remains promising but immature. Although advances in sequence-level engineering of PET hydrolases continue, industrial tolerance to pigments, multilayer adhesives, and food-contact residues remains under-evaluated [59,156,168]. Consequently, the scalability of biological conversion remains conditional on purification logistics, energy decarbonization, and robust enzyme-stability models.

A structural modification persists in the governance and traceability of recycled materials. Many countries lack integrated digital platforms that authenticate material custody, verify recycled content, and assign economic value according to process history [116,154]. This is particularly evident in regions where informal labor sustains the majority of material recovery. In Latin America, Southeast Asia, and parts of Africa, informal pickers deliver >60% of post-consumer plastic feedstock, yet the lack of traceability undermines fair payment and limits compliance verification [148,154,169]. Experience from Malaysia demonstrates that when externalities are internalized—rather than evaluating only direct costs—economic rankings of waste-management options are reversed, revealing that incineration is systematically loss-inducing. At the same time, mechanical recycling generates net positive externalities [144]. The implications indicate that circular-economy policy must deploy full-system costing to avoid distorted technology prioritization.

AI emerges as one of the strongest corrective instruments for these gaps. Its influence is visible across operational, design-level, and governance layers. At the operational level, machine-learning-based process control has demonstrated reductions of 20–40% in pyrolysis energy demand through temperature-yield optimization, catalyst-selectivity prediction, and real-time parameter adjustment [85,160]. In mechanical systems, AI-enabled classification has reduced reject rates by up to one-third. At the same time, predictive analytics have been used to anticipate reductions in washing water and stabilizer consumption, as well as MFI-dependent failure modes [138,167]. These applications convert uncertainty into actionable optimization and materially shift recovery-to-waste ratios.

At the material design level, AI-driven surrogate modeling enables virtual screening of formulations before pilot-scale testing. Molecular-level prediction of tensile modulus decay, crystallinity loss, and long-term aging kinetics now enables high-value upcycling strategies to be evaluated computationally, reducing physical testing cycles and R&D emissions footprints [160,167,171]. Likewise, integrating machine-learning-assisted TEA-LCA forecasting enables scenario-dependent environmental planning, in which variations in electricity decarbonization, infrastructure scaling, or substitute-grade performance are evaluated before deployment [171].

From a governance and enforcement perspective, AI is enabling stronger compliance frameworks through digital signature-based traceability, automated quality scoring, and blockchain-linked certificate auditing [163,164]. These conclusions should be interpreted within explicit system-boundary choices; as summarized in Section 5.1 and Table 3, multi-cycle modeling, open-loop cascades, and regionalized logistics/energy mixes can materially shift LCA/TEA outcomes and therefore the recommended routing of plastic streams.

Overall, future circular-plastic architectures will require more than technical optimization—they must align environmental accounting, digital traceability, operational feasibility, and social inclusion. The case evidence demonstrates that systems achieve durable performance only when policies ensure regulatory continuity, market incentives secure stable demand for recycled materials, and digital systems authenticate real-time material flows [153,167]. Under these conditions, artificial intelligence shifts from a supplemental optimization instrument to a structural enabler of transparency, comparability, and performance assurance. Without continued convergence of AI-supported platforms, standardized LCAs, and inclusive labor frameworks, circularity gains will remain partial and technologically fragile rather than systemic.

## 9. Conclusions

Circular plastic systems are undergoing a structural transition driven by the convergence of artificial intelligence (AI), advanced recycling technologies, and sustainability-oriented governance. Across mechanical, chemical, biological, and upcycling pathways, the reviewed evidence indicates that AI is evolving from an auxiliary analytical tool into a key enabling layer for predictive decision-making, improved system efficiency, and higher-value circular outputs. AI strengthens material quality assurance through intelligent sorting, enhances process performance by anticipating degradation and reaction behavior, and improves environmental planning through TEA–LCA-informed forecasting under realistic substitution and energy-integration scenarios.

However, persistent systemic gaps remain. Recycling outcomes continue to depend strongly on feedstock variability, degradation accumulation, and insufficient traceability across supply chains. Many LCAs still rely on mass-based functional units that do not capture durability, barrier performance, or service-life equivalence of secondary materials. Likewise, biological and thermochemical routes show promise but require technological maturation, energy decarbonization, and validated catalyst/enzyme stability to achieve scalable performance.

The cases analyzed further demonstrate that circularity is not solely technology-dependent; it is reinforced by policy continuity, full-system costing of externalities, and the inclusion of informal labor systems that sustain recovery in many regions. Harmonized regulatory frameworks—particularly those integrating digital tracking, quality standards, and EPR-linked incentives—emerge as critical enablers of stable secondary-material markets.

Overall, future circular plastic systems will be defined by (i) AI-enabled diagnostic and decision infrastructures; (ii) validated substitution performance of secondary materials; (iii) low-carbon integration of emerging recycling routes; (iv) governance models ensuring transparency and equitable value distribution. Under these conditions, circularity becomes technically viable, environmentally consistent, and economically competitive, enabling comparable assessments and robust scaling across regions and supply chains.

## Figures and Tables

**Table 1:** Comparative framework of mechanical, chemical, and biological recycling routes, summarizing the core process, significant operational constraints, and typical product outputs.

Mechanical	Chemical	Biological
Extrusion process Compatibilization Thermal stabilization	Pyrolysis, solvolysis Gasification	Enzymatic hydrolysis Microbial conversion
	Catalytic cracking	Chemo-bio hybrids
Optimization by: Screw profile Residence time Real-time ML	Optimization by: Catalysis formulation Kinetic control Energy integration	Optimization by: Enzyme affinity Crystallinity reduction Pathway redesign
Key constraints: Degradation of physical properties	Key constraints:	Key constraints:
	High energy	Slow kinetics
	Complex mixtures	Lack of datasets
Typical outputs: Regranulate for high-substitution products (e.g., rigid packaging/non-food contact goods, crates/pallets, construction profiles/pipes)	Typical outputs:	Typical outputs:
	Monomers/hydrocarbons	Monomers/intermediates
	Decision-support point (TEA+LAC+IA-based yield prediction) Maximized circular value	

**Table 2:** Representative applications of AI tools in plastic upcycling, highlighting optimization strategies, target processes, and performance outcomes across chemical, biological, and materials-design pathways.

Authors	Application of AI	Results
Wang et al. [87]	SERDA, SVR, PSO, XGBoost	Optimization of pyrolysis catalytic conditions to maximize fuels
Wang et al. [87]	Predictive models	Transformation of medical waste into activated carbon with high CO_2_ capture performance
Huang & Lee [88]	Structural optimization	Production of electrodes from PP mask
Cui et al. [89]	Algorithm-assisted design	Selective photocatalysts for depolymerization
Rezaei et al. [90]	Practical bioinformatic	Prioritization of metabolic pathways for biodegradation
Li et al. [91]	Neural Networks	Porous carbon optimization with maximum CO_2_ sorption

**Table 3:** System boundaries applied to LCA of circular plastics, functional purpose, and representative literature.

System Boundary	Description	Typical Use Case	Representative Literature
Cradle-to-Gate	Covers the transformation from raw feedstock or collected waste to the production of usable recycled resin	Comparative assessments of mechanical vs. chemical vs bioconversion technologies; benchmarking efficiency of pyrolysis units	Huang et al. [55]; Jeswani et al. [57]; Chen et al. [106]
Gate-to-Gate	Focused exclusively on intra-plant processes such as washing, extrusion, catalytic depolymerization, pelletization	Optimizing specific unit operations, industrial diagnostics, and energy allocation analysis.	Martínez-Narro et al. [27]
Cradle-to-Grave	Includes production, use, and end-of-life scenarios, including landfill disposal, incineration, and recycling.	Packaging evaluations; comparisons of polymer alternatives for equivalent protection performance.	Jeswani et al. [57]; OECD [107]
Cradle-to-Cradle	Incorporates full circular feedback; recovered product reenters the system with a virgin equivalent	“Bottle-to-bottle” PET system; chemical recycling routes returning monomer substitutes	Uekert et al. [56]; Tang et al. [99]
Multi-cycle cradle-to-cradle (dynamic/cascaded)	Includes 2–N cycles with degradation/quality and allocation/substitution rule.	Scenarios where “how many cycles are beneficial” or quality limits are evaluated.	Guinée et al. [100]
Open-loop cascade (cradle-to-cascade/open-loop)	Recycled material falls into another functional category (downcycling); requires explicit FU and substitution.	Mixed PE/PP ending in profiles, street furniture, construction, etc.	Schyns & Shaver [22], Grant et al. [109]
Regionalized cradle-to-gate (with transport & energy mix)	Includes interregional logistics and local energy mix (electricity/heat), infrastructure and regional EoL charges	transboundary waste/regranulate chains; Local vs. Exported Processing Comparisons	Lin et al. [110]; van Schaick [111]; Montag [112]

## Data Availability

No new data were created or analyzed in this study.

## Abbreviations

The following abbreviations are used in this manuscript:

AI	Artificial Intelligence
CE	Circular Economy
CED	Cumulative Energy Demand
GEP	Global Warming Potential
HDPE	High-density polyethylene
LCA	Life Cycle Assessment
LICA	Life Cycle Impact Assessment
LCI	Life Cycle Inventory
LDPE	Low-density polyethylene
ML	Machine Learning
MFA	Material Flow Analysis
MRFs	Material Recovery Facilities
MSW	Municipal Solid Waste
P-graph	P-graph Model
PHOENIX	PHOENIX Framework
PE	Polyethylene
PET	Polyethylene terephthalate
PLA	Polylactic Acid
PP	Polypropylene
PS	Polystyrene
PVC	Polyvinyl chloride
RDF	Refuse-Derived Fuel
TEA	Techno-Economic Analysis
